# The Cross-Modal Effects of Sensory Deprivation on Spatial and Temporal Processes in Vision and Audition: A Systematic Review on Behavioral and Neuroimaging Research since 2000

**DOI:** 10.1155/2019/9603469

**Published:** 2019-12-02

**Authors:** Laura Bell, Lisa Wagels, Christiane Neuschaefer-Rube, Janina Fels, Raquel E. Gur, Kerstin Konrad

**Affiliations:** ^1^Child Neuropsychology Section, Department of Child and Adolescent Psychiatry, Psychosomatics and Psychotherapy, Medical Faculty, RWTH Aachen University, Aachen, Germany; ^2^Department of Psychiatry, Psychosomatics and Psychotherapy, Medical Faculty, RWTH Aachen University, Aachen, Germany; ^3^JARA-Brain Institute I, Brain Structure Function Relationships (INM-10), Research Center Juelich, Juelich, Germany; ^4^Clinic of Phoniatrics, Pedaudiology and Communication Disorders, Medical Faculty, RWTH Aachen University, Aachen, Germany; ^5^Teaching and Research Area of Medical Acoustics, Institute of Technical Acoustics, RWTH Aachen University, Aachen, Germany; ^6^Department of Psychiatry, Perelman School of Medicine, Philadelphia, PA, USA; ^7^Lifespan Brain Institute, Children's Hospital of Philadelphia and Penn Medicine, Philadelphia, PA, USA; ^8^JARA-Brain Institute II, Molecular Neuroscience and Neuroimaging, RWTH Aachen & Research Centre Juelich, Juelich, Germany

## Abstract

One of the most significant effects of neural plasticity manifests in the case of sensory deprivation when cortical areas that were originally specialized for the functions of the deprived sense take over the processing of another modality. Vision and audition represent two important senses needed to navigate through space and time. Therefore, the current systematic review discusses the cross-modal behavioral and neural consequences of deafness and blindness by focusing on spatial and temporal processing abilities, respectively. In addition, movement processing is evaluated as compiling both spatial and temporal information. We examine whether the sense that is not primarily affected changes in its own properties or in the properties of the deprived modality (i.e., temporal processing as the main specialization of audition and spatial processing as the main specialization of vision). References to the *metamodal organization*, *supramodal functioning*, and the *revised neural recycling theory* are made to address global brain organization and plasticity principles. Generally, according to the reviewed studies, behavioral performance is enhanced in those aspects for which both the deprived and the overtaking senses provide adequate processing resources. Furthermore, the behavioral enhancements observed in the overtaking sense (i.e., vision in the case of deafness and audition in the case of blindness) are clearly limited by the processing resources of the overtaking modality. Thus, the brain regions that were previously recruited during the behavioral performance of the deprived sense now support a similar behavioral performance for the overtaking sense. This finding suggests a more input-unspecific and processing principle-based organization of the brain. Finally, we highlight the importance of controlling for and stating factors that might impact neural plasticity and the need for further research into visual temporal processing in deaf subjects.

## 1. Introduction

*Neural plasticity* refers to the capability of the brain to adjust and reorganize its neural structure. One of the most remarkable instances of these adaptive changes occurs following *sensory deprivation* [1, 2]. The recruitment of the visual cortex of blind individuals during auditory tasks has functional relevance and actively contributes to the enhanced auditory spatial performance (e.g., see [3]). Thus, the ability of the brain to adapt its neural functions and structures ensures that brain regions that are usually responsible for processing signals from the deprived sense are still used. In deaf and blind individuals, the effects of sensory deprivation are often investigated in terms of the *cross-modal* takeover of the lost sensory functions [4–6]. Here, *cross-modal plasticity* refers to the recruitment of cortical regions and functions of the lost sense by other remaining senses. Two opposing views emerged to account for the effects of sensory deprivation on the remaining senses. The *perceptual deficit hypothesis* states that the deprivation of one sense results in deficiencies in the remaining modalities/senses [7], such as impaired vertical auditory localization following blindness [4]. In contrast, according to the *sensory compensation hypothesis*, the deprivation of a sense generates “above-normal” effectiveness of the remaining modalities with an improvement in their functional capabilities [7]. For example, deafness leads to enhanced visual performance in the periphery that is accomplished *inter alia* by auditory cortex activation [8]. Findings from previous studies are inconsistent, and a dichotomous view of either enhancements or deficits might be too simplified [9, 10].

**Figure 1:**
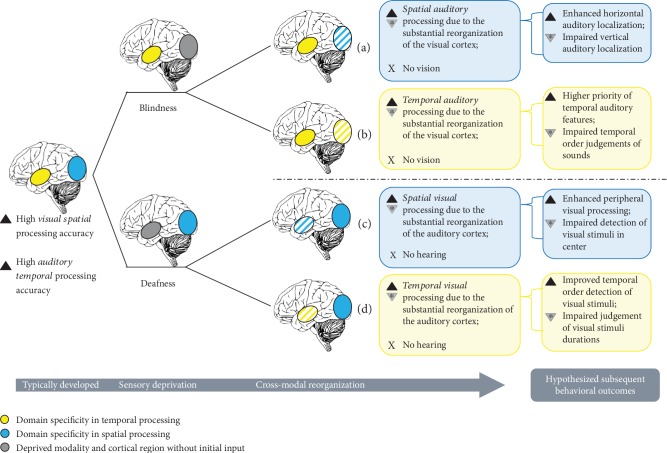
Illustration of the possible consequences of cross-modal reorganization following sensory deprivation. In typically developed individuals, the occipital cortex executes the highly detailed spatial processing of visual stimuli, whereas the temporal cortex is adequately used for the high temporal processing of auditory stimuli. Following blindness, (a) an improvement in spatial auditory processing abilities and/or (b) an improvement in temporal auditory processing abilities occurs through the takeover of certain regions in the occipital cortex (hatched area). Following deafness, (c) an improvement in spatial visual processing abilities and/or (d) an improvement in temporal visual processing abilities occurs through the takeover of certain regions in the temporal cortex (hatched area). Notably, outcomes (a) and (b), as well as (c) and (d), are not necessarily mutually exclusive. Additionally, certain aspects of sensory processing are plausibly decreased after cortical reorganization, as indicated by the asterisks. Hypothesized subsequent behavioral outcomes are shown on the right side of the figure.

Previous reviews addressed various effects of neural plasticity in blindness or deafness (e.g., [9, 11–16]). The current review uses a new approach that compares spatial and temporal visual/auditory processing in deaf and blind individuals, respectively. Thereby, we examine whether cross-modal plasticity is mainly associated with improved or deteriorated performance in aspects in which the deprived or overtaking sense specializes. The human brain consists of neural systems supporting different functions required to process sensory information. Pascual-Leone and Hamilton [17] described the brain as *metamodal.* Senses compete for processing in different regions, depending on the demands of the sensory modalities. Cortical areas are not solely specified for the processing of one sense. However, a domain-specific dominance of the respective senses has been observed. Within this framework, the visual system provides highly spatial information. The occipital cortex is the most adequate region for processing highly detailed spatial aspects and topographical mapping of the environment [17]. Hearing, on the other hand, enables the detection of the temporal order of auditory events with high precision through the use of various auditory cues (i.e., the interaural time and level differences, as well as spectral cues between both ears) and the tonotopical representation in the auditory cortex [18, 19]. Importantly, when deprived of one sense, which is consequently no longer the winning competitor, cortical regions are generally able to process information derived from other senses [17]. Accordingly, spatial and temporal processes represent two of the most important operating principles of the visual and auditory cortices. The current review will focus on both spatial and temporal processing principles in blind and deaf individuals to compare the effects of visual and auditory deprivation, respectively. Considering the metamodal organization and the initial specialization of the senses, the question arises as to which functions are primarily affected by neural plasticity. The following hypotheses are derived by relating this principle to the two opposing views of enhancements or deficits occurring following sensory deprivation:

1. Recruitment of the cortical area associated with the deprived sense (i.e., in blindness, the occipital cortex for vision, and in deafness, the temporal cortex for audition) results in superior performance in its initial specialization:

- Auditory spatial processing will be enhanced in blindness (Figure 1(a))
- Visual temporal processing will be enhanced in deafness (Figure 1(d))

(2) Recruitment of the cortical area associated with the deprived sense results in superior performance in the specialization of the overtaking sense:

- Auditory temporal processing will be enhanced in blindness (Figure 1(b))
- Visual spatial processing will be enhanced in deafness (Figure 1(c))

Figure 1 depicts an overview of the derived hypotheses of the possible consequences of blindness or deafness, based on the assumption that cortical reorganization occurred. A hypothesized subsequent behavioral outcome is provided for each hypothesis. Notably, the hypotheses are not mutually exclusive. Therefore, neural plasticity following sensory deprivation might result in enhancements and/or deficits in the temporal and/or spatial domain of the overtaking sense. Furthermore, while senses have an initial specialization, all senses operate together and form our perceptions through the integration of multisensory information. Decreased perceptual abilities in blind or deaf individuals might therefore result from a lack of scaffolding, which is usually provided by the deprived sense (Figure 1, indicated by an asterisk (∗)).

**Figure 2:**
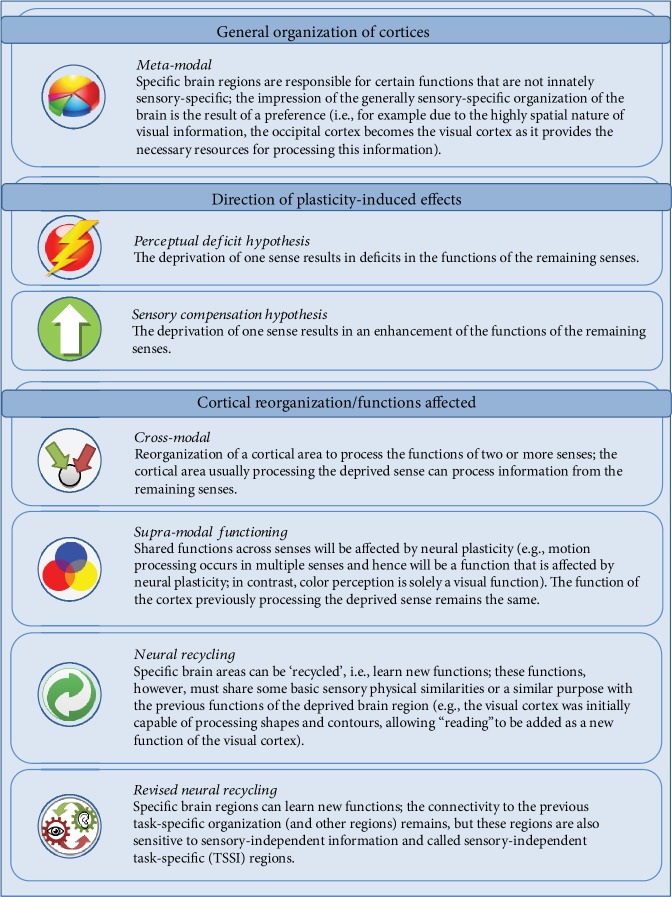
Theories about brain organization and neural plasticity. Theories are outlined and categorized as general cortical organization principles, directional hypotheses of the neural plasticity-induced effects and theories that state which functions are likely affected. A further description of the theories and a reference to corresponding publications is provided in the main text.

Researchers examining deaf cats proposed a theory for plasticity-related reorganization principles that limits the functions affected by plasticity [20, 21]. Shiell et al. [21] describe the *supramodal function theory* to account for deafness as follows: (i) cross-modal plasticity only occurs for functions that are apparent in more than one sensory modality, e.g., movement detection; (ii) the function of the cortical module remains the same; and (iii) cross-modal plasticity is limited to functions that are supported by the auditory cortex. This theory presupposes an overlap between the functions that are lost due to deprivation and functions that are provided by the overtaking sense. For example, compared to hearing cats, deaf cats showed superior visual movement detection. The superior performance was causally related to the cortical area that typically processes auditory motion, thus confirming cross-modal reorganization [20]. Recent evidence obtained from humans suggests a task-selective and sensory-independent reorganization of brain areas during cross-modal plasticity [15, 22]. This reorganization might be viewed as an extension of the *supramodal functioning hypothesis* addressed in human research. Briefly, the *neural recycling* term was introduced by Dehaene [23] and refers to the takeover of preexisting brain systems by novel functions. The novel function must share some basic *sensory-specific* features with the previous specialization of the sensory region of the brain, allowing the novel functions to be consolidated. Further, the takeover is limited by evolutionary anatomical and connectivity constraints, and this prior evolutionary organization is never entirely overwritten [23, 24]. Thus, *neural recycling* was initially thought to occur only within the same sense. The *revised neural recycling theory* proposed by Amedi et al. [15] together with Dehaene [23, 25] adapted this idea, noting that these shared features are not required to be sensory-specific. Accordingly, (i) a connection from the cortical region of the deprived sense to its larger network that is task-specific remains intact, and (ii) sensory-unspecific areas are located within this cortical region, which are labeled as task-selective sensory-independent (TSSI) areas [15, 24, 25]. An example of a TSSI brain organization would be the recruitment of the voice perception area in the temporal cortex of deaf individuals during face perception. The prior task specialization of identity representation of the auditory cortex is thus retained and now similarly utilized for vision [26]. See Figure 2 for an overview of all previously reported theories and cortical reorganization principles of neural plasticity.

Relating these reorganization principles to the hypotheses formulated above, the current systematic review has two main aims. First, regarding the metamodal organization principle and the prior specialization of each sense, we will explore whether specializations of the deprived or overtaking sense are primarily altered (Figure 1). Second, we will systematically analyze whether the TSSI reorganization principle is validated for tasks involving spatial or temporal processing in blind and deaf individuals. Therefore, the current review will focus on the spatial and temporal auditory abilities of blind individuals, as well as the spatial and temporal visual abilities of deaf individuals. An overview of past behavioral and neuroimaging studies of vision and audition investigating a cross-modal takeover will be provided using the structure of the hypotheses derived above (see also Figure 1). Movement detection tasks that require the combination of both temporal and spatial processing strategies are addressed to complement the findings. Finally, similarities and implications for future investigations and clinical interventions are discussed. Thus, we summarize basic processing principles in blind and deaf individuals and recent theories on the organization of the brain to provide a behavioral and neural framework of basic plasticity-related changes based on human research conducted since 2000.

## 2. Methods

### 2.1. Systematic Search Strategy

The PubMed database was searched using single keywords and keyword combinations of the abstract/titles of the available articles listed in Supplementary [Supplementary-material supplementary-material-1]. The search was always initiated by paired keywords to ensure that the selected articles addressed either the auditory abilities of blind individuals or the visual abilities of deaf individuals. The records retrieved from the database search were further screened according to the following criteria: The articles were written in “English,” published between January 2000 and December 2018, and examined human subjects only. Papers were excluded if they examined tinnitus or genetic syndromes (e.g., Usher, Alström, or Alport syndrome). The search conducted in December 2018 revealed 2,482 articles. During the first screen, duplicates and articles addressing any other medical or mental conditions other than deafness and/or blindness were removed. The first evaluation yielded 531 articles of possible relevance, including six articles identified through cross-referencing.

### 2.2. Study Selection and Data Management

**Figure 3:**
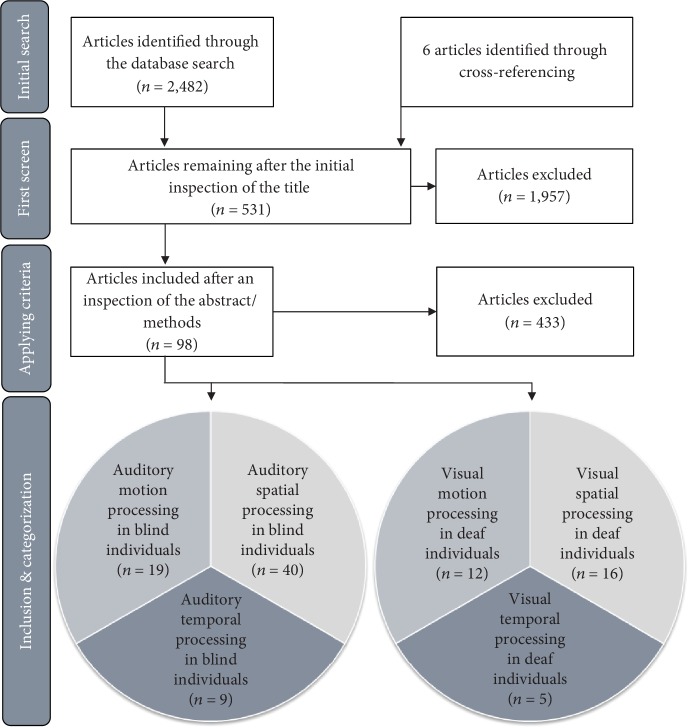
Flow diagram of the systematic search strategy. The search strategy and selection of the articles are illustrated as a stepwise process.

The abstracts of those 531 articles were screened. For inclusion, studies were required to address either the spatial or temporal auditory abilities of blind individuals, the spatial or temporal visual abilities of deaf individuals, or visual/auditory motion processing. Whenever an abstract did not contain sufficient information, the methods, in particular the tasks assigned, were evaluated. Importantly, studies addressing language or speech processing, as well as word recognition, were excluded. Higher-order processing involving language and memory was not in the scope of the current review. Controlling for language is particularly difficult, since previous studies rarely assessed the language experience and proficiency of participants or compared deaf children born to deaf parents with deaf children born to hearing parents. The importance of language experience is stressed elsewhere (e.g., see a comment by MacSweeney and Cardin [27]). We did not restrict our review to studies including either congenitally blind or deaf subjects only but included subjects with all conditions associated with vision or hearing loss at any time during life. Furthermore, neuroimaging studies were included only if an active response was required during the task, due to the combined interest in the cross-modal effects on the neural networks and behavior. However, volumetric and connectivity analyses were reviewed to complement the findings. Finally, articles addressing sensory restoration, as revealed by the current literature search, were included to provide a detailed overview. However, sensory restoration is a very complex topic that has not been extensively investigated herein and was more exhaustively discussed by, e.g., Heimler et al. [9]. Similarly, intracortical alterations are considered to be linked to behavioral alterations reported in behavioral studies but are beyond the scope of the current review. The main focus was on the basic processing principles of vision and audition to maintain a clear comparison between the two senses and to limit other possible influences. This selection process identified 98 relevant articles for the current review. An overview of the study selection procedure is presented in Figure 3. Notably, articles can be included in more than one category if they examined spatial and temporal processing.

## 3. Adaptation to Visual Deprivation

### 3.1. Auditory Spatial Processing in Blind Individuals (Figure 1(a))

Due to visual deprivation, blind individuals rely strongly on their remaining senses to orient in space and to navigate through their environment. Consequently, numerous studies have addressed the question whether blind individuals exhibit altered spatial localization abilities in the remaining senses. Referring to Figure 1, this implies that most experimental studies have investigated whether hearing (one remaining sense) improved in the domain (spatial processing) in which the deprived sense (vision) was initially specialized (see Figure 1(a)). These investigations rely mainly on daily life experiences and case reports, showing that some blind individuals are capable of using echolocation for navigation [28–30]. Tasks administered to assess spatial localization abilities vary broadly and affect performance outcomes. When considering behavioral studies conducted with blind individuals (without any neural measures), it is important to note that the reported behavioral adaptations might be attributed to cross-modal alterations. Nevertheless, it cannot be precluded that intracortical changes and/or alterations in the sensory organs (e.g., the retina) are similarly linked to these behavioral changes.

#### 3.1.1. Behavioral Level

While no differences in accuracy were observed when assessing *auditory spatial attention* in blind individuals, faster reaction times recorded during selective (and divided) attention paradigms suggest generally enhanced spatial attention [31, 32]. When elaborating *spatial localization*, one must essentially differentiate between two scenarios. When referring to an external frame of reference, the participant must judge whether a sound is located on the right or the left side of a reference sound, i.e., an external reference point (relative distance judgment). When internal reference (i.e., egocentric) frames are applied, a subject must point towards a sound source using his or her own body as reference point (absolute distance judgment). However, pointing to an object with the index finger is not necessarily an ecologically valid method of responding by an individual who is blind. Studies addressing the horizontal plane predominantly concluded that blindness does not affect absolute binaural sound localization, as evidenced by subjects pointing towards the sound source [33, 34]. Rather, it led to improved monaural horizontal sound localization abilities [4, 35]. Experience and a more sophisticated use of spectral, echo, and distance cues were speculated to account for the improved localization abilities, mainly in the horizontal plane [36–38]. The head position exhibited special relevance to blind individuals when assessing the position of sounds relative to a specific location in the azimuth, i.e., in a horizontal circle around the individual. Specifically, audiomotor feedback might calibrate the auditory space [39] such that the spatial perception of blind individuals becomes more body-centered. The ego-center of blind individuals indeed was shown to be closer to the center of head rotation than that of sighted individuals [40]. In contrast, studies investigating the vertical plane often describe a deficit in sound localization [4, 41, 42]. Again, the frame of reference is decisive. Some individuals exhibited a deficit in the absolute localization of sounds in the vertical plane [42]. However, when asked to indicate the location of a sound relative to another source, some blind individuals did not differ in accuracy from sighted individuals [42]. Interestingly, the simultaneous investigation of monaural and binaural, as well as vertical and horizontal sound localization, showed that the decrease in sound localization abilities in the vertical plane is due to a trade-off. Individuals with a deficit in vertical sound localization were generally those with heightened horizontal localization abilities [4].

*Spatial coding* mechanisms of hearing may explain the observed trade-off in blind individuals. In contrast to the place coding used by other senses, the population coding mechanism in audition is proposed to consist of two neural populations, one for the left hemispheric field and the other for the right hemispheric field [18]. Therefore, only a minor proportion of neurons represents the frontal field [18]. This left/right hemispheric coding might additionally explain why only the horizontal and predominantly peripheral sound localization, but not the vertical localization, was enhanced in blind individuals. Studies investigating left/right discrimination often report a superior performance of blind individuals in a variety of tasks, such as echolocation or sound source discrimination (e.g., see [36, 37, 43]). Echolocation experts discriminated the horizontal auditory space with a similar acuity range as the horizontal visual space is discriminated in sighted individuals [44]. Vision mainly provides important feedback for auditory localization during vertical sound localization and sound localization in the frontal field [41]. It is thus thought to serve as a reference for the spatial processing of information obtained from the other senses [45]. Within this context, sighted individuals benefit from multisensory integration to accurately localize objects within the auditory space and strongly rely on vision. However, when comparing blind with sighted individuals, many studies blindfolded the sighted participants or asked them to close their eyes while investigating auditory localization (e.g., see [46] or [38]). The auditory localization of sighted individuals was affected by the blindfold in all room dimensions, with the localization of the horizontal plane noted as particularly poor [35, 47].

In addition to horizontal and vertical localization, *distance/spacing* constitutes a further important spatial feature. Blind compared to sighted individuals showed worse relative auditory distance judgments involving sounds presented in the front [48]. Using references in the extrapersonal space, they overestimated distances nearby and underestimated distances farther away [49]. Similarly, when investigating auditory distance abilities by spatial bisection, i.e., judging whether a sound was spatially closer to the first or the third sound of a three sound sequence, the spatial localization of blind individuals was decreased (e.g., see [45, 50, 51]). Blind echolocation experts, however, were able to train their spatial bisection abilities [50]. Again, task design is relevant. Whereas blind individuals performed poorly in judging distances using references in the external space (relative discrimination), they showed superior performance in paradigms that use body-centered reference frames (absolute discrimination) [38, 52]. However, blind children and children and adults with low vision did not have such superior distance discrimination abilities [38]. This finding suggests that some improvements in these skills are attributed to exposure and training.

Similarly, *minimal audible angle tasks* are frequently employed to assess spatial abilities. In these tasks, participants must decide whether a sound is coming from the left or the right side of a central source location while the angle between the sounds varies. Compared to sighted individuals, blind individuals showed equal or better performance on these tasks [45, 46, 50, 53]. However, blind individuals appeared to use different localization strategies, e.g., facing the objects with the ear [53]. Strikingly, in one study, *late* as well as *early* blind individuals outperformed sighted individuals in determining relative positions and distances. This implies that some supranormal performance can occur even after late sensory loss [46].

Finally, uniting all spatial aspects, *self-localization* is highly dependent on the remaining senses when an individual is visually deprived. Studies addressing self-localization/obstacle circumvention indicate that early blind individuals show superior localization performances [33, 46, 54, 55]. A potential explanation is the substantial reliance of blind individuals on auditory cues to navigate through their environment and the superior use of echolocation to localize and detect obstacles (e.g., see [28, 56]). Notably, the degree of vision loss was linked to spatial accuracy [57]. The earlier and more pronounced the visual loss, the higher the spatial accuracy.

#### 3.1.2. Neural Level

Several studies identified a link between enhanced behavioral performance and visual cortex recruitment. Based on electroencephalography (EEG) investigations, congenitally blind individuals display greater accuracy when *localizing sounds* [58, 59]. This superior behavioral performance was accompanied by a posterior shift in the EEG components N1 and P3, representing a stimulus feature-processing and attentional marker, respectively. Interestingly, in addition to the recruitment of the visual cortex, the frontal eye fields, which are usually associated with oculomotor processes, showed functional relevance for spatial auditory attention, although voluntary eye movements are irrelevant in blind individuals. The stimulus-oriented activation of these areas possibly reflects the preparation of head movement [60], supporting the notion that audiomotor feedback might calibrate auditory spatial perception. Furthermore, functional magnetic resonance imaging (fMRI) investigations similarly revealed activation within the occipital cortex of early blind individuals. In particular, the right cuneus and middle occipital gyrus, part of a network underlying visuospatial processing, were activated during *monaural localization* (e.g., [61], [62] or [60]). Cortical thickness was linked to the functional activation of the occipital cortex in blind individuals [61, 63, 64]. Specifically, stronger activation within the visual cortex was associated with thinner cortical structures, likely reflecting the specialization of these areas through pruning [63]. Moreover, an increased coherence between frontocentral and occipital regions was observed [58, 59].

Although increased activation of the right occipital cortex is thus often reported, increased behavioral performance is not always found (e.g., [65]). Nevertheless, the increased occipital cortex recruitment during auditory spatial tasks correlated with increased performance (e.g., [62]). Fundamentally, visual cortex regions were only activated in those individuals showing superior performance. A study employing transcranial magnetic stimulation (TMS) provides similar evidence of the functional importance of this cross-modal plasticity [3]. Specifically, when TMS was applied over the right occipital cortex, the performance of blind subjects decreased. Notably, when TMS was applied over the right intraparietal sulcus, which commonly processes auditory spatial information, early blind individuals did not display impairments in their spatial localization abilities [3]. This compensatory effect of activation of the right middle occipital gyrus and cuneus was observed in *early* blind, but not *late* blind, individuals [64]. Although alterations in occipital cortex recruitment have been detected in late blind individuals, their effects on behavioral performance appear less straightforward [66]. Rather, activation of the right ventral occipital cortex in late blind individuals correlated with poorer performance [67]. These findings suggest a cortical area-specific time period during which these regions are recruited to perform compensatory functions during auditory spatial processing.

Finally, virtual acoustics, i.e., the simulation of sound appearing from different locations played via headphones, enables investigations of the underlying neural mechanisms in more natural paradigms. It provides a possibility to circumvent the limitations derived from some imaging techniques, such as fMRI. However, no behavioral differences were reported in the few studies using virtual acoustics, although, again, early blind individuals recruited posterior parietal areas and the (right) middle occipital gyrus for sound localization, with the latter being related to performance [63, 65, 68]. The investigation of two *echolocation* experts revealed that activation of the calcarine cortex while listening to virtual (echo) vocalizations provided information about the spatial origin (left/right) of the auditory stimulus [69]. This finding supports the functional relevance of the recruitment of visual cortical regions for auditory spatial processing following blindness after experienced usage of echolocation.

### 3.2. Auditory Temporal Processing in Blind Individuals (Figure 1(b))

In contrast to the well-studied spatial auditory abilities of blind individuals, less evidence for similar effects on temporal processing abilities is available (see Figure 1(b)). Presumably, this lack of information is attributable to the fact that hearing is a sense that is already initially capable of processing temporal aspects with greater precision than that of vision. Hence, temporal order judgments are rarely assessed, and reaction times are the main focus of studies assessing the auditory temporal abilities of blind individuals.

#### 3.2.1. Behavioral Level

Within the context of *reaction time assessments*, blind individuals did not only exhibit a higher sensitivity in terms of accurate location detection within the periphery but also reacted faster to stimuli presented in the periphery and in the frontal visual field [70]. Moreover, compared to sighted individuals, blind individuals did not react slower to stimuli in the periphery than the frontal field [70]. The question arises whether this generally faster reaction to auditory stimuli results from better spatial localization, enhanced temporal processing, or a combination of the two. Better estimates of temporal processing performance might be revealed by tasks that address *duration or asynchrony* and *temporal order judgments* of visual stimuli. Although early blind individuals' performance in discriminating durations and detecting gaps did not appear to be enhanced [71, 72], thresholds for asynchrony detection and temporal order judgments were significantly lower than those for sighted individuals [71, 73].

#### 3.2.2. Neural Level

In contrast to the behavioral study by Lerens et al. [71], who did not observe improved *duration discrimination* in early blind individuals, an EEG study reported faster and more accurate responses of blind subjects during duration discrimination. These responses were accompanied by an enhanced negative signal (N1) in the occipital cortex [74]. Similarly, a previous study showed a link between improved performance in auditory *temporal order judgments* and occipital cortex activation [75]. Given the high precision of temporal auditory processing in a normally developing individual (e.g., [76]), congenitally blind individuals might favor temporal rather than spatial stimulus selection strategies. This would provide support for the hypothesis that hearing (the remaining sense) is indeed enhanced in functions that are already specialized (temporal domain; see Figure 1(b)). An investigation of both the spatial and temporal processing abilities of blind individuals showed that blind individuals indeed rely more on temporal than spatial aspects when selecting a stimulus [77]. Enhanced N1 ERP activation during temporal, but not spatial, *stimulus selection strategies* in blind individuals supports this notion [77].

### 3.3. Auditory Motion Processing in Blind Individuals (Figures 1(a) and 1(b))

In real world environments, auditory stimuli often are not static but rather move in space and change dynamically. Without visual input, blind individuals must rely more on auditory motion localization, namely, the ongoing encoding of temporally ordered spatial auditory cues.

#### 3.3.1. Behavioral Level

The importance of vision in *auditory motion detection* became apparent in studies comparing children with low vision to children with total blindness and with late and early blind adults [78, 79]. Blind children performed worse or equal to children with low vision in static and dynamic (horizontal and vertical) sound localization tasks. The *level of remaining vision* was linked to better performance. Adult-like performance was achieved at approximately the age of 13 years [78]. The worse performance of blind children and equal performance of *early* and *late* blind adults suggests that experience and strong reliance on nonvisual cues are responsible for the behavioral advantage observed in a variety of auditory motion detection/lateralization tasks (e.g., see also [80] or [81]). Interestingly, the abilities of early blind individuals to detect auditory motion were affected by head motion [82]. These individuals showed a leftward bias during static sound detection and a slight leftward bias during moving sound detection. Moreover, the localization was biased towards the direction of the head movement. Thus, blindness results in more body-centered spatial representations [82] instead of the visuomotor loops that might assist in auditory motion processing in sighted individuals [41, 80, 83].

Using a 2-dimensional setup requiring the *reproduction of the sound movement*, complex aspects of sound motion localization abilities were assessed in a recent study [83]. Depending on the continuous encoding of sound positions, early blind individuals were less accurate when detecting sound motions in the lower plane. Late blind participants did not show this impairment, and neither late nor early blind individuals exhibited impairments in horizontal sound motion localization. An insufficient development of audiospatial maps, with which vision might assist in sighted individuals, might underlie this phenomenon.

Even after cataract treatment and *sight recovery*, effects of auditory motion on visual motion perception persisted (i.e., an optical illusion during which long exposure to moving auditory stimuli affects a stationary visual stimulus that is consequentially perceived as moving) [84]. Neither sighted nor visually impaired individuals showed this effect. Thus, despite early sight recovery, i.e., in infants at ages ranging from 5 to 24 months, cross-modal changes persist long after restoration of sight.

#### 3.3.2. Neural Level

Despite the lack of a behavioral difference between blind and sighted individuals in two-alternative forced choice tasks assessing *movement differentiation*, early blind individuals showed activations in visual (motion) areas (V1/V2/V3 and the middle temporal complex (hMT+), including the middle temporal (MT) and medial superior temporal (MST) visual areas) [85–88]. The literature is not univocal about the question whether sound motion is solely processed in areas that are responsible for processing visual motion in sighted individuals. Further, whereas early blind individuals displayed reduced functional connectivity between the MT/MST and other visual regions, connections between the MT/MST and frontal regions were strengthened [85]. Interestingly, some visual motion areas were recruited by blindfolded sighted individuals [89], supporting the hypothesis of a generally sensory-independent organization of the brain.

Similarly, studies assessing auditory *motion detection* have not provided unequivocal answers to the question whether only visual motion-processing areas are recruited for auditory motion processing. An investigation of event-related potentials assessing performance during motion detection observed the recruitment of motion-related and nonmotion-related visual areas [90]. It was suggested that enhanced performance of early blind individuals is thus not exclusively supported by the visual areas required for motion processing alone [90]. In contrast, in a functional imaging study, by simulating azimuthal movement through changes in sound intensity, the activation pattern was restricted to the right occipitotemporal regions that process motion [91]. The functional specialization of this region thus remained unchanged and served auditory instead of visual motion processing in blind individuals. Although no behavioral difference was observed, preexisting connections between auditory and visual cortices might be strengthened to support auditory motion processing in blind individuals [91]. In fact, a discrimination between blind and sighted individuals was possible based on differing activation patterns of hMT+ (activated in blind individuals) and the right planum temporale (activated in sighted individuals) during auditory motion processing [92].

Notably, while this additive shift was apparent across a variety of tasks, it often only applied to *early* and not *late* blind individuals [86, 93]. Late blind individuals did not recruit the hMT+ and did not lose the activation pattern in the right planum temporale [92, 93]. Compared to early blind individuals, late blind individuals also did not outperform sighted individuals in an auditory motion task [92, 93].

Moreover, neural plasticity related to *echolocation-based motion* appears to differ from general auditory motion [30]. While blind and sighted individuals performed equally well, echolocation experts exhibited superior echolocation-based motion detection. The superior performance of blind echolocation experts in echolocation tasks may result from experience and therefore an improved analysis of the temporal information provided (e.g., by the echo following the self-produced click in a relatively short period of time). Importantly, temporal-occipital cortical areas responsible for echolocation differed from areas recruited for processing sound motion [30].

Finally, consistent with the results of the behavioral studies, cross-modal neural adaptations appear to occur, even during short phases of congenital blindness, followed by (partial) *sight recovery*, and can coexist with the regained visual functions [93, 94]. After sight recovery, the visual motion area hMT+ was recruited for visual and auditory motion detection [84, 93]. A recent electrophysiological study revealed worse performance on a visual global motion task (i.e., detecting the global motion of visually presented dots) for participants whose cataracts were reversed than that for sighted and visually impaired individuals. However, these individuals outperformed the other two groups in an auditory global motion task [95]. This finding was similarly reflected in the oscillatory brain activity recorded during both tasks. The sensory modalities were not postulated to compete for the same neural resources, and the impaired visual functioning was mainly attributed to the lack of early visual input [95].

### 3.4. Consequences of Visual Deprivation

Taken together, the direction of the effect of neural plasticity strongly depends on the experimental setup. As previously suggested [96], the spatial dimensions of the room and the frame of reference represent crucial factors affecting neural plasticity. The most frequently investigated function in blind subjects is spatial processing, the main specialization of vision (Figure 1(a)). Here, the most consistent findings were the enhancements in monaural horizontal sound localization (in the periphery), self-localization, and echolocation. Prominently, these changes were observed when the body was used as the reference frame. However, the vertical sound localization and distance discrimination of blind individuals were impaired. Primarily, those individuals who showed an improvement in horizontal localization exhibited poor vertical localization. The use of an external frame of reference negatively affected performance. Although only a few studies have directly investigated temporal auditory processing (Figure 1(b)), faster reactions to peripheral visual stimuli represent another prominent finding. However, reaction times are not a direct measure of temporal auditory processing *per se*. Further tasks assessing temporal order judgments, duration detection, or discrimination are warranted to more directly address temporal auditory processing in blind individuals. Similar to spatial auditory processing, enhancements and deficits in auditory motion processing have been observed (Figures 1(a) and 1(b), comprising spatial and temporal processing). Whereas blind individuals showed improved horizontal motion detection, sound localization in the lower visual plane was impaired. A leftward bias and a bias in the direction of head movement indicate a higher reliance on body-centered spatial references. Notably, changes induced by cross-modal plasticity appear to be driven by experience, as mainly observed in subjects with congenital blindness, and persist even after sight recovery.

**Figure 4:**
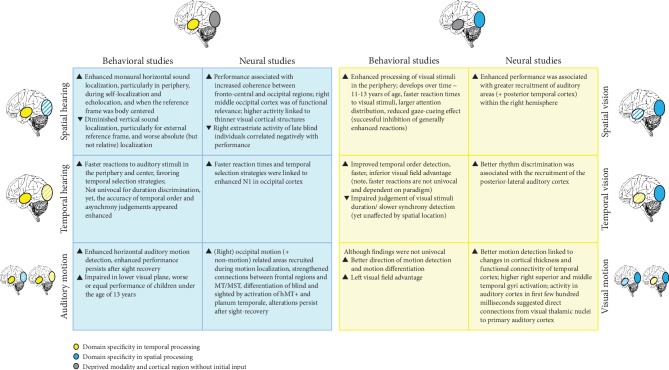
Consequences of visual and auditory deprivation. Summary of the behavioral and neural results of the reported studies addressing visual and auditory deprivation. The major findings for spatial, temporal, and movement processing are depicted separately, and the hypotheses listed in Figure 1 are referenced. In blind individuals, (a) improvements/impairments in spatial auditory processing abilities and (b) improvements/impairments in temporal auditory processing abilities are observed. In deaf individuals, (c) improvements/impairments in spatial visual processing abilities and (d) improvements/impairments in temporal visual processing abilities are observed. The hatched areas illustrate the possible nature of the improvement (temporal/spatial). However, the alterations reported in behavioral studies might not only result from cross-modal plasticity but also intracortical changes and alterations within the sensory organs. The respective studies are reported in the main text, and a detailed overview is provided in Supplementary [Supplementary-material supplementary-material-1].

Corresponding neural studies often linked the increased visuospatial performance (Figure 1(a)) to task-specific regions within the occipital cortex, particularly the middle occipital gyrus. The recruitment of the middle occipital gyrus during auditory spatial tasks has been confirmed in various fMRI, TMS as well as virtual acoustics studies. Since the middle occipital gyrus is associated with visual spatial processing, this finding is consistent with the *revised neural recycling theory* and hence TSSI reorganization [15]. In addition, a strengthened connectivity between frontal/central and occipital areas supports the engagement of this region in spatial auditory tasks. The timing of sensory deprivation, however, appears crucial for the direction of neural effects induced by cross-modal plasticity. In late blind individuals, greater activation of the right ventral visual pathways during sound source discrimination was negatively correlated with performance. Furthermore, imaging research on temporal auditory processing capabilities in blind individuals (Figure 1(b)) is sparse and information about behavioral temporal processing is mainly derived from reaction times only. Nevertheless, associations between improved task performance and neural activation in the visual cortex have been reported, e.g., linking temporal selection strategies to increased occipital activity. The recruitment of the (right) visual (motion) area during auditory motion localization similarly supports the hypothesis of compensatory neural plasticity following blindness (Figures 1(a) and 1(b), comprising spatial and temporal processing). This cross-modal plasticity-related enhancement was again only observed in early and not late blind individuals. Interestingly, the recruitment of visual motion brain areas persisted after sight restoration, although these regions were now recruited during visual movement detection. After sight recovery, individuals still showed superior performance in auditory motion detection but worse performance than that of sighted individuals during visual motion detection. Finally, comparable to spatial auditory localization, strengthened connections between frontal and visual motion processing areas were linked to enhanced behavioral performance. See Figure 4 for an overview of the findings and Supplementary [Supplementary-material supplementary-material-1] for a detailed description of the reported studies.

## 4. Adaptation to Auditory Deprivation

After considering both spatial and temporal processing in blind individuals, we will now elaborate plasticity-related alterations in visual spatial and temporal processing occurring following auditory deprivation. Importantly, similar to the behavioral results obtained from blind individuals, the reported changes in performance in studies examining behavior alone (without any neural measures) might be due to cross-modal alterations, as well as intracortical changes and/or alterations within the sensory organs. Additionally, most studies related to deafness have investigated perceptual abilities after hearing restoration through cochlear implantation and (dis)advantages of cross-modal plasticity that affect or even prevent hearing rehabilitation (e.g., for a recent review, see [97]) rather than simple spatial and temporal processes. Compared to blind individuals, although sight restoration is possible in some cases, the restoration of hearing via cochlear implantation is implemented more frequently. The restoration of sight appears to be much more difficult, as the neural representation of the visual world is more complex than the encoding principles used in auditory processing (for a comparison of sensory restoration, see [9]).

### 4.1. Visual Spatial Processing in Deaf Individuals (Figure 1(c))

Nonetheless, visual spatial processing in deaf individuals (Figure 1(c)) has also been extensively investigated. This might be explained by the notion that visual spatial information is very important when a person is unable to hear.

#### 4.1.1. Behavioral Level

Previous studies concluded that deafness leads to an increased sensitivity of spatial processing abilities in the periphery [98, 99]. Inferences about improved visual spatial abilities in deaf individuals highly rely on differences in reaction times [76, 100]. Notably, *spatial attention* is one major concept related to visual spatial processing. It is a multifaceted ability that has been investigated in deaf individuals using various tasks. We will first address neural plasticity during childhood and adolescence, followed by neural plasticity related to visual spatial processing in adults.

Visual attention appears to develop differently in hearing and deaf children [101–104]. A comparison of the performance of deaf and hearing children (5-15 years of age) on *visual detection* tasks revealed reaction time impairments in young deaf children [99]. Young deaf individuals showed slower reaction times and reduced accuracy during the detection of light stimuli in the far peripheral field. At the age of 13-15 years, deaf children outperformed hearing children when detecting certain visual stimuli in the periphery. It was postulated that deaf children might undergo a longer period of cross-modal reorganization and redirection of attention to the periphery [99]. Similar studies were conducted by applying different visual tasks related to spatial attention in children [101–104]. The age at which an increase in performance was reported varies. When assessing *visual selective attention*, deaf children (7-17 years of age) only outperformed hearing children after 11 years of age [101]. No differences were observed in *visual sustained attention* [102]. However, young deaf children were more likely to be distracted by peripheral information than hearing children (before the age of 9) [102], and at ages of 5-12 years, deaf children used slower *visual search* strategies [103]. Notably, the selection criteria for participants in the deaf population play a major role in determining the effects, particularly regarding the degree of hearing loss/auditory simulation. This was confirmed in a study comparing children who received a cochlear implant (CI) and used oral language with children without a CI who mainly utilized sign language [104]. While visual alerting was impaired and executive functioning was not affected in response to low auditory stimulation compared to high auditory simulation, two orienting mechanisms, moving and engaging, were enhanced and lead to faster orienting [104]. Thus, the high reliance on visual experiences, including sign language, likely shapes the behavioral performance of deaf children.

Similarly, outcomes of studies with deaf adults vary depending on the age range, stimuli, and task administered. For example, a study in young adults (18-40 years of age) was unable to replicate a previous investigation showing enhanced spatial performance in the periphery in participants aged greater than 13 years [101, 105]. However, the task instructions differed and were more focused on the central targets. The attentional focus is thus decisive when investigating spatial processing. Additionally, investigations in the *near* and *far visual field* should be differentiated. The near field has been investigated more frequently. Overall, it was posited that deafness leads to a greater focus of attention within the peripheral field. This result has been observed in studies using various visual attention paradigms, as deaf individuals show higher interference by peripheral distractors or faster reactions to stimuli within the periphery [106–108]. Sign language alone is not sufficient to induce these changes [107, 108]. Although hearing signers display enhanced performance compared to hearing nonsigners, deaf individuals exhibited even shorter reaction times when detecting visual stimuli at various locations in a far peripheral forced choice paradigm [108]. An increased attention to the periphery was similarly observed in studies investigating visual selective attention using a Flanker paradigm. In the near peripheral field, deaf adults were more affected by visual distractor stimuli than hearing adults. Interestingly, in the far space, the performance of deaf and hearing individuals was comparable in the periphery, whereas the performance of deaf participants was impaired in the center [98]. Deaf individuals likely allocated their spatial attention to a wider range, explaining the higher interference in Flanker paradigms [98, 109]. A *line bisection* paradigm similarly suggests a wider attention distribution in deaf individuals. Whereas hearing individuals appeared to have a leftward line bisection bias in the spatial distribution of visual attention, deaf signers and deaf nonsigners showed no bias towards any spatial hemisphere [110]. Notably, other investigations found that deaf adults orient or react slower, yet less erroneous, to stimuli compared to hearing adults (e.g., [109] or [111]). Different task designs and responses requested (i.e., ocular response vs. head turn) might underlie the heterogeneity. Finally, while deaf and hearing adults showed comparable arrow cueing effects, i.e., invalidity effects during reorienting of attention tasks with exogenous cues, deaf individuals were less affected by *gaze cueing* than hearing individuals [112]. Gaze cueing refers to how gaze affects participants' attention towards a specific target. The reduced gaze cueing effect probably endows deaf individuals with better spatial attention to faces and might result from top to down experience-dependent plasticity. Hence, in addition to previously reported findings indicating changes in bottom-up attentional aspects, top-down attentional components might be similarly altered [112]. Comparably, top-down visuospatial processes were investigated by assessing *eye movements* in deaf individuals during an overt saccadic target-selection task, i.e., when searching for a target between stimuli and a distractor target [113]. Interestingly, deaf adults showed slower saccadic responses, which in turn most likely produced a diminished saliency effect. That is, saliency manipulations by color affected responses of deaf individuals to a lesser extent (colored target, colored distractor, or no-color changes). Again, this suggests that these generally faster responses can be successfully inhibited. For a further overview of visual attention paradigms used in deaf individuals and how the selection of individuals and tasks affects outcomes, see reviews by Dye and Bavelier [114] and Tharpe et al. [115].

#### 4.1.2. Neural Level

Studies investigating the link between altered *visuospatial localization* in deaf individuals and neural mechanisms are sparse. One investigation linked neural mechanisms to enhanced performance in the periphery [6]. Specifically, mainly right hemispheric cortical activity in higher auditory processing regions (i.e., Brodmann area 22, a posterior temporal cortex region) was associated with improved performance (i.e., lower detection thresholds and hence faster localization of targets) and to simultaneous differences in the activation of visual areas. Importantly, the enhanced behavioral performance is only detected in the peripheral task with distractors. It was argued that the lack of difference in the performance of hearing and deaf participants on the peripheral localization task without distractors might be due to the stimuli used.

### 4.2. Visual Temporal Processing in Deaf Individuals (Figure 1(d))

Compared to various behavioral studies addressing the spatial (attention) domain, only a few studies have investigated temporal visual processing in deaf individuals (Figure 1(d)), with a focus on the temporal order perception and temporal duration discrimination.

#### 4.2.1. Behavioral Level

The accuracy of detecting the correct visual *temporal order* was comparable in deaf and hearing individuals [76]. However, faster reactions of deaf individuals were observed when detecting the *asynchrony* of visual stimuli [76]. This advantage for deaf individuals was particularly pronounced when the first stimulus appeared in the peripheral field and under the condition with the shortest asynchrony between the two visual stimuli [76]. Similarly, deaf individuals were more accurate or performed equally well as hearing individuals during *synchronized finger tapping* to visual stimuli [116]. When judging the synchrony of light stimuli, the reaction times of deaf individuals were slower than those of hearing individuals, regardless of location [117]. Notably, deaf individuals showed comparable visual temporal thresholds, regardless of whether stimuli were presented in the central or peripheral field, whereas the performance of hearing controls was affected by location [117]. The latter result is consistent with the findings of speeded/simple detection tasks that do not require spatial localization or temporal discrimination. Here, deaf individuals appear to exhibit faster responses, regardless of the location of the target (i.e., periphery or center) [118, 119]. This implies an altered attention distribution rather than temporal processing *per se*.

In addition to the altered visual attention distribution, some deficits in auditory temporal duration perception were reported following deafness. Deaf individuals' *estimation of the duration* of a visual stimulus, by reproduction of the stimulus duration, was impaired [120]. This finding is consistent with the auditory scaffolding theory [121]. Audition is hypothesized to be superior in temporal and sequential processing, providing a scaffolding mechanism for other senses. Consequentially, when individuals lack this aid, other senses are impaired in the processing of temporal aspects. In alignment with this theory, deaf children experienced difficulties in number processing but did not show a deficit in spatial but rather temporal order processing/serial recall [122].

#### 4.2.2. Neural Level

Similar to the auditory studies conducted in blind individuals, many neural studies investigating deafness did not report any behavioral differences. Technical issues or task selection might have influenced the results [8, 123]. Additionally, the auditory cortex, i.e., Heschl's gyrus, varies widely among subjects [8]. Imaging studies that do not consider this difference might underestimate the cortical changes within the auditory cortex that potentially follow auditory deprivation. After considering between subject variability, regions of the primary auditory cortex and supramodal and multisensory cortical areas were altered in deaf participants [8]. Further, in a recent study, deafness led to the processing of *visual rhythm discrimination* in the auditory cortex [22]. Specifically, the brain regions recruited by deaf individuals during visual rhythm discrimination in the posterior-lateral section of the auditory cortex resembled the area that the hearing individuals recruit during a similar auditory task [22]. Although the generally faster response to visual stimuli is often understood as an indication of improved temporal processing abilities, higher speed does not necessarily indicate enhanced temporal processing abilities *per se*. Instead, it might also be related, for example, to differences in (connectivity to) motor areas. In this context, it is important to mention that enhanced reactivity has been linked to intracortical (auditory cortex) changes [100].

### 4.3. Visual Motion Processing in Deaf Individuals (Figures 1(c) and 1(d))

Another insight into possible temporally and spatially related behavioral and cortical changes is provided by studies investigating visual motion processing in deaf individuals. The temporal judgment of moving objects might be more relevant in daily life situations, e.g., when catching a ball or evading a moving obstacle, rather than determining the temporal order of an event with high temporal accuracy. Studies investigating visual motion processing might thus delineate a distinct natural testing environment.

#### 4.3.1. Behavioral Level

Intuitively, deaf individuals would be equipped with better visual motion processing to compensate for the loss of auditory information that might have provided assisting information. Surprisingly, in early investigations, deaf individuals did not outperform hearing individuals in *motion detection* [124]. However, in a later study, deaf individuals were faster and more accurate during *motion localization* and the detection of the direction of motion [125]. Similarly, lower thresholds were observed when detecting moving visual stimuli (in the periphery) [21, 126]. These inconsistencies might be explained by task-specific differences. For example, when comparing visual motion detection in the horizontal and vertical planes, compensatory plasticity was only observed for horizontal motion detection [127]. Additionally, after applying different versions of the random-dot task, a left visual field advantage in deaf individuals was revealed during movement localization [125]. The finding of the left visual field advantage appears to contradict the findings reported in other studies. A right hemisphere advantage was frequently reported for deaf individuals and linked to sign language abilities [128, 129]. An older age at which the participants acquired signing might explain the lack of a right visual field advantage [125]. Disentangling the differing visual field advantages was enabled in an investigation of hearing nonsigners, deaf signers, and hearing signers [128, 130]. The peripheral visual motion processing improvement and the left visual field advantage during visual motion detection were linked to deafness. The right visual field advantage was attributed to sign language experience [128]. However, most studies investigated individuals born in deaf families. These individuals are exposed to sign language early in life. It should be kept in mind that these individuals represent a minor proportion of the deaf population [131].

#### 4.3.2. Neural Level

Structural changes, such as changes in cortical thickness or myelination, as well as alterations in functional connectivity during the passive observation of visual motion might be linked to better motion detection [132–134]. However, only a few studies have explored the link between these alterations and behavior during a visual motion detection task. Magnetoencephalography (MEG) performed during *visual motion discrimination* revealed the occurrence of auditory cortex activation in deaf individuals in the first few hundred milliseconds. This suggests the presence of a direct projection from the visual thalamic nuclei to the primary auditory cortex [135]. Further, as a key region involved in multimodal integration, the postsuperior temporal sulcus (STS) is likely affected by sensory deprivation. STS activity was increased in early deaf individuals [131]. In particular, the right superior and middle temporal gyri were involved in *motion detection* [136, 137]. Notably, motion-related brain regions showed increased activation in deaf but not hearing signers and thus likely represent functional plasticity due to auditory deprivation and not signing [131]. Finally, additional cortical changes were observed in regions of the posterior parietal cortex, which largely support selective attention. This result highlights the importance of changes other than cross-modal changes and potentially explains the selective attention shift reported following auditory deprivation [131].

### 4.4. Consequences of Auditory Deprivation

Most studies explored the consequences of auditory deprivation on spatial processing (Figure 1(c)), i.e., the specialization of the overtaking sense. Notably, the variety of different tasks, particularly the variety of spatial visual attention paradigms, might have contributed to the heterogeneity of findings from previous studies. Nevertheless, one of the most frequently reported findings is the enhanced processing of visual stimuli in the periphery. However, enhanced performance in spatial visual processing appeared to be mainly driven by experience. Increased visual attention in the periphery (in the near and far visual fields) only became apparent from approximately 11 to 13 years of age. The use of sign language did not explain these enhancements. The second prominent finding, again comparable to blind individuals, is the faster reaction to visual stimuli. Moreover, deaf individuals successfully inhibited these generally enhanced reactions to visual stimuli, which might be particularly important during social interactions. Deficits in visual spatial performance were mainly reported in young deaf children as well as during temporal visual processing. While deafness resulted in improved temporal order detection and temporal synchronization, performance in the judgment of visual stimulus duration was impaired (Figure 1(d)). Peripheral and horizontal visual motion detection and differentiation were improved in deaf individuals, particularly in the left visual field (Figures 1(c) and 1(d), comprising spatial and temporal processing). Importantly, many studies relied only on reaction times. Although reaction times might be considered an indicator of changes in temporal processes, when spatial processing is not involved in the task being investigated, reaction times should not be regarded as a sensible measure for temporal processing *per se*. Further studies investigating visual temporal and motion processing that do not solely rely on reaction time measures are warranted.

Few imaging studies have linked the enhanced performance of deaf individuals on spatial visual processing in the periphery (Figure 1(c)) to the recruitment of the right auditory cortex. In addition, findings suggest the recruitment of auditory brain regions responsible for rhythm discrimination during visual rhythm discrimination (Figure 1(d)). This supports the hypothesis that task-selective, yet sensory-independent brain regions are affected by cross-modal plasticity, as stated in the *revised neural recycling theory* [15]. Importantly, enhancements in temporal processing appear to be linked to intracortical rather than cross-modal changes. Finally, alterations in the activity of the auditory cortex in the first few hundred milliseconds likely support visual motion discrimination (Figures 1(c) and 1(d), comprising spatial and temporal processing). Figure 4 provides a brief summary of the findings, and Supplementary [Supplementary-material supplementary-material-1] contains a detailed description of the reviewed studies.

## 5. Discussion

The aim of the current review was to identify whether cross-modal plasticity is linked to enhancements or deficits in processes in which the deprived and/or the overtaking sense specializes. Furthermore, we investigated whether these alterations fit into the framework of previously proposed reorganization principles.

### 5.1. Common Findings from Blind and Deaf Individuals

Some similarities become apparent between visual and auditory deprivations. First, in both types of sensory deprivation, enhanced processing of peripheral stimuli has often been reported. This finding contrasts results observed in sighted and hearing individuals who mainly favor the central field. Nonsensory-deprived individuals likely utilize the integration of information from multiple senses to efficiently perceive the entire spatial environment [70]. This was supported by studies showing that a blindfold impaired the performance of sighted individuals during auditory tasks [35, 47]. Next, although developmental studies are not available for all reviewed aspects, experience and the age at which a sensory modality is lost appear to play a crucial role in behavioral adaptations. Analogously, most studies agree that neuronal reorganization is the most prominent and produces the most beneficial changes in individuals with total and congenital deprivation compared to the minor and late loss of sensory function. Interestingly, right hemispheric regions are frequently recruited following cross-modal plasticity in blind and deaf individuals. The functions within the cortical areas responsible for processing information from the deprived sense and the overarching task circuits remain intact when recruited by another sense. However, the neural takeover is limited by the capacities of the overtaking sense (e.g., auditory cues do not work as efficiently in the frontal visual field, particularly in the vertical plane, as vision). Finally, when the deprived sense is restored, i.e., sight and hearing recovery, changes in behavioral and neural plasticity remain intact, despite the (re)gained ability to process the deprived sense.

Taken together, the spatial domain (specialization of vision, Figures 1(a) and 1(c) is the aspect that has been most frequently investigated for both types of sensory deprivation. Most likely, this is due to the fact that all senses collectively provide the resources to experience our surroundings. Sensory deprivation limits the advantage of multisensory integration and hence limits the rich spatial representation of the environment. Although most research of deafness and blindness reveals enhanced performance in spatial processing, further research in the temporal domain is warranted. Future studies should explore the temporal domain alone or in combination with various spatial dimensions to answer the fundamental question which temporal abilities are enhanced or attenuated following blindness and deafness. An overview and comparison of the aforementioned reported changes within each sensory deprivation are presented in Figure 4. The findings are assigned to the hypotheses introduced and summarized in Figure 1. Moreover, we concluded that the findings from the reviewed investigations are highly consistent with the *revised neural recycling theory*, which hypothesizes that the brain is organized in a TSSI manner [15, 24, 25]. Importantly, recent human research supported the hypothesis that this organization principle occurs in the auditory in addition to the visual cortex, in which it has been more frequently reported (e.g., [22] and [26]).

### 5.2. Remarks and Clinical Implications

Some remarks about these investigations of neural plasticity and their clinical implications are necessary. First, multiple terms for the same or similar phenomena have been used in previous studies of cross-modal plasticity. For example, the *perceptual deficit hypothesis* is comparable to the (sensory) deficiency/loss theory/hypothesis or general loss hypothesis [10, 138, 139]. The *sensory compensation hypothesis* is similar to the compensatory adaptation theory [139]. Alternatively, these phenomena are also referred to as adaptive/compensatory or maladaptive changes in plasticity or enhancements and deficits, respectively [9, 114]. Generally, the theories on plasticity-related reorganization principles are semantically very similar. See Figure 2 for clarification.

Next, although the cross-modal takeover appears to follow strict functional rules that are consistent with the theories described in this review, these findings should not be translated to each case of sensory loss. Multiple factors impair the generalization of the outcomes and make cross-modal plasticity a highly individualized phenomenon. Thus, caution is warranted when comparing various studies of neural plasticity. On a fairly basic level, for example, plastic reorganizations of the brain due to damage or deprivation of a sense might largely differ from adaptations of the brain caused by experience and the learning of new skills. Various forms and degrees of plasticity have been observed in previous studies [1, 140–142], which may serve separate functions. Importantly, plastic changes occurring after sensory deprivation are not exclusively cross-modal neural changes. Alterations might similarly occur in intracortical areas [143] and in the sensory organ itself [144]. Although these changes have not been extensively investigated herein and were beyond the scope of the current review, they are an important aspect to consider when interpreting the plasticity-related changes reported in behavioral and neural studies. An overview, yet not exhaustive list, of several types of plasticity and influencing factors is presented in Table 1. Hence, although plasticity mechanisms work in a specific manner, the question whether it is adaptive or maladaptive can hardly be generalized.

Moreover, several processing differences exist in the visual and auditory systems [9]. This implies that the neural takeover is limited to certain processes and might not entirely be covered when referring to vision and hearing alone. This becomes apparent when examining shared sensory functions of hearing and touch and changes in somatosensory processing after deafness [151, 152]. Notably, somatosensory processing was not included in the current systematic review, as its organization is somatotopic and naming a single main specialization for touch appears less straightforward (for the multiple concepts of touch, see, e.g., [153]). Critically, the extent of the somatosensory deprivation varies substantially with regard to the body parts and sensations [153]. Most importantly for the current review, classifying touch as spatial and temporal processing, as performed here for vision and audition, would be too simplified. Nevertheless, distinct parts of the deprived cortices are likely to be recruited by different overtaking senses. For example, Ricciardi et al. [154] address this issue in a meta-analysis of tactile and auditory processing in blind individuals and Striem-Amit et al. [155] investigated compensatory plasticity in people born without hands.

Although not all adaptations following cross-modal plasticity are either advantageous or disadvantageous, the reviewed findings may be helpful for future investigations or interventions. For example, noninvasive neural stimulation, techniques to enhance neural plasticity, and advantageous behaviors might be most effective in studies focusing on brain areas and connectivity involved in task-specific processes. Similarly, when developing sensory-substitution devices, i.e., the compensation for sensory loss by another sense through technical devices (e.g., hearing through a device that translates speech into tactile patterns), the notion of spatial and temporal processing enhancements in blind and deaf individuals, respectively, might guide technical developments to obtain suitable devices assisting individuals with tasks in daily life. Future virtual acoustic reality investigations might assist in realistic explorations of plasticity-induced effects (e.g., see [156] or [157] for further examples of virtual acoustic realities). This might be particularly useful for imaging studies assessing auditory tasks, enabling investigations of more complex tasks instead of simple assessments of two alternative forced choices. Finally, using functional near-infrared spectroscopy (fNIRS) to investigate changes pre- and postimplantation of a CI, the effects on behavior and neural organization of a CI itself (neural rehabilitation) could be further disentangled from the neural reorganization attributed to auditory deprivation (e.g., for first initiatives using fNIRS, see [158] or [159]).

## 6. General Conclusions

The systematically outlined behavioral and neural findings provide a new framework to investigate how specific aspects of sensory processing are altered in blind and deaf individuals. Importantly, a clear overlap of the consequences of auditory and visual deprivation was observed. Various investigations primarily revealed alterations in spatial processing, allowing enhanced perception of the spatial environment after sensory deprivation. Future research investigating temporal auditory processing in blind individuals and temporal visual processing in deaf individuals are warranted to obtain a complete picture of the rules shaping cross-modal plasticity. Generally, behavioral performance is adapted in processes for which the overtaking and the deprived modality provide adequate resources. A modification mainly in the peripheral field and the right hemisphere of the brain becomes apparent. Concisely, these findings support a more sensory-unspecific but task- and principle-organized structure of the brain, which persists after sensory deprivation. This framework will likely be of high relevance for the development of sensory substitution devices and future investigations utilizing noninvasive brain stimulation.

## Figures and Tables

**Table 1:** Types of plasticity and influencing factors.

Type	Function
(1) Strengthening of cognitive functions	Skill learning (e.g., [145])
(2) Hemispherectomy	Removal of one hemisphere to treat a variety of seizure disorders, leading to a takeover of functions that were initially performed by or in combination with the removed hemisphere (e.g., [146])
(3) Sensory substitution	Compensation of sensory loss by another sense or external device (e.g., [147]); for a review addressing differences within hearing restoration by cochlear implantation, see [97]
(4) Early deprivation	Early loss due to a genetic or medical condition leading to compensation and broad takeover by other senses, although functional topography appears inert as dual streams (dorsal and ventral) remain intact; reorganization mainly occurs through bottom-up processing (e.g., [148, 2])
(5) Late deprivation	Rather supportive in nature; compensation for the loss is restricted due to initial pruning and functional reorganization; rather through top-down processes (e.g., [148, 2])
(6) Site of plastic changes	Cross-modal, intracortical, or even within the sensory organ (e.g., the retina [144])

Influencing factors	
(1) Sensitive/critical periods	
(2) Other senses and their critical periods [149]	
(3) Age of onset of deprivation	
(4) Duration of deprivation	
(5) Degree of loss [142]	
(6) Cause of sensory deprivation	
(7) Working memory, intelligence quotient, gender (…) (e.g., see also the Ease of Language Understanding (ELU) model [150])	

## Supplementary Materials

Supplementary 1 Table S1: keywords and paired keywords for the systematic literature review.

Supplementary 2 Table S2: overview of included studies per sensory deprivation and spatial/temporal process.
