# Supplementary material for: The Cross-Modal Effects of Sensory Deprivation on Spatial and Temporal Processes in Vision and Audition: A Systematic Review on Behavioral and Neuroimaging Research since 2000
Source: Neural Plast. 2019 Dec 2;2019:9603469. doi: 10.1155/2019/9603469 (PMC6914961; doi:10.1155/2019/9603469)
Supplement: Supplementary 1 — Table S1: keywords and paired keywords for the systematic literature review. [file 9603469.f1.pdf]

Supplementary Material

Table S1. Keywords and paired keywords for the systematic literature review.

Impairment/Plasticity term		Addition
Cochlear implant	AND	Vision
Cochlear implant	AND	Visual
Hearing impairment	AND	Visual
Hearing impairment	AND	Vision
Deaf	AND	Seeing
Deaf	AND	Visual
Deaf	AND	Vision
Auditory deprivation	AND	Vision
Deafness	AND	Visual
Auditory impairment	AND	Visual
Auditory impairment	AND	Vision
Hearing aid	AND	Vision
Hearing aid	AND	Visual
Cataract	AND	Hearing
Blind	AND	Hearing
Blind	AND	Auditory
Visual deprivation	AND	Hearing
Blindness	AND	Hearing
Visual impairment	AND	Hearing
Blind	AND	Temporal cortex
Blind	AND	Auditory cortex
Hearing	AND	Occipital cortex
Hearing	AND	Visual cortex
Deaf	AND	Occipital cortex
Deaf	AND	Visual cortex
Cortical reorganization	AND	Deaf
Cortical reorganization	AND	Blind
Sensory deprivation	AND	Plasticity
Crossmodal reorganization		
Cross modal reorganization		
