# Supplementary material for: The Cross-Modal Effects of Sensory Deprivation on Spatial and Temporal Processes in Vision and Audition: A Systematic Review on Behavioral and Neuroimaging Research since 2000
Source: Neural Plast. 2019 Dec 2;2019:9603469. doi: 10.1155/2019/9603469 (PMC6914961; doi:10.1155/2019/9603469)
Supplement: Supplementary 2 — Table S2: overview of included studies per sensory deprivation and spatial/temporal process. [file 9603469.f2.pdf]

Supplementary Material

Table S2: Overview of included studies per sensory deprivation and spatial/temporal process.

Study – Author(s)	Blind (B)/ Deaf (D)	Spatial/ Temporal/ Motion	Imaging technique/ Behavioral only	Blind/ Deaf participants (Age; onset of blindness/ deafness)	Task(s)	Results: Between group differences
Abel et al. [35]	B	Spatial	Behavioral	30-55 years of age; 9 participants were congenitally blind or blind before 3 years of age and 6 got blind after 10 years of age	Horizontal sound source identification using an 8 loudspeakers-array manipulating auditory information	Late blind individuals performed better than congenitally blind as well as blindfolded control participants, increase in number of loudspeakers led to decrease in accuracy
Cappagli et al. [48]	B	Spatial	Behavioral	Blind children 9-17 years of age, blind adults 20-72 years of age; 10 children and 7 adults (4 early and 3 late blind)	Auditory spatial (distance) localization (23 loudspeakers in one line in front not horizontal arc): 2 alternative forced choice: which source is closer to oneself	Blind subjects performed worse than sighted, however late blind individuals' performance was not impaired
Chen et al. [43]	B	Spatial	Behavioral	22 ± 1.8 years of age; Individuals that were blind: Experiment 1: 14, Experiment 2: 16, Experiment 3: 15 (no details - short report)	Sound presented, then sound again either at same or different location (to left or right of mid-sagittal plane) or same or different frequency: Experiment 1: detect presence of the target; Experiment 2: localize (left vs. right) the target; Experiment 3: discriminate high low frequency	No behavioral difference (might be due to generally low error rates), congenitally blind localized peripheral sounds faster than sighted, but this was not followed by changes in attention-orienting mechanisms; enhanced auditory where, yet impaired what pathway processing was observed in blind individuals
Collignon et al. [32]	B	Spatial	Behavioral	19-67 years of age; 8 early blind individuals, congenitally blind (1 lost vision at 18 months)	Localization of left vs. right (auditory and tactile; selective and divided attention), choice reaction time task and simple reaction time task	No performance difference in sensory sensitivity/simple reaction time task, blind individuals showed faster reaction times during selective spatial (and bimodal) attention task

Study – Author(s)	Blind (B)/ Deaf (D)	Spatial/ Temporal/ Motion	Imaging technique/ Behavioral only	Blind/ Deaf participants (Age; onset of blindness/ deafness)	Task(s)	Results: Between group differences
Collignon and De Volder [31]	B	Spatial	Behavioral	20-56 years of age; 8 congenitally blind individuals	Localization of left vs. right (auditory and tactile; selective and divided attention), choice reaction time task and simple reaction time task	Similar accuracy but faster reaction times of blind individuals in selective (left/right) (and divided attention) task
Després et al. [57]	B	Spatial	Behavioral	9 congenitally blind: 18-58; 11 late blind: 20-60; 5 amblyopic: 22-60, 4 near-sighted: 20-55 years of age	Self-positioning in room by listening to auditory cues: 2 repetitive sounds played 5 times from each of 8 loudspeakers, assess position on plan drawn on sheet of paper	Early blind individuals performed better than late blind, amblyopic and near-sighted individuals, extent of spatial accuracy related to degree of visual loss
Despres et al. [54]	B	Spatial	Behavioral	18-47 years of age; 7 congenitally blind individuals	Self-localization task (simple acoustics: successively played sounds, complex acoustics: simultaneously played sounds; 8 loudspeakers positioned in semicircular manner)	Blind individuals were better in self-localization than sighted individuals in simple and complex acoustic environment
Despres et al. [33]	B	Spatial	Behavioral	19-36 years of age; 6 congenitally blind individuals	Spatial localization and attention orienting (covert orienting, auditory uninformative cues) and the role of eye movement on localization (8 loudspeakers positioned in semicircular manner)	Effect of eye movement preserved in early blindness, equal performance in localization in frontal hemi-field, faster reaction times of blind individuals when sound in far-lateral position, no difference in effect of attentional cues between blind and sighted
Doucet et al. [34]	B	Spatial	Behavioral	20-71 years of age; 10 blind individuals: 8 congenitally, 1 at 8 years of age and 1 at 14 years of age	Monaural and binaural sound localization (16 loudspeakers – semicircular perimeter)	Blind subjects performed well in binaural sound localization, half of the blind participants were able to localize under monaural condition, those were retested, and the spectral cues were altered: errors increased when ability to use spectral cues was altered

Study – Author(s)	Blind (B)/ Deaf (D)	Spatial/ Temporal/ Motion	Imaging technique/ Behavioral only	Blind/ Deaf participants (Age; onset of blindness/ deafness)	Task(s)	Results: Between group differences
Dufour et al. [37]	B	Spatial	Behavioral	18-55 years of age; 6 congenitally and 6 late blind individuals (blind between the age of 13 and 40)	Echolocation (right/left) and simple azimuthal localization (2 different condition: distance two lateral walls varied)	Blind individuals (early and late) showed enhanced sensitivity to echo cues and outperformed sighted individuals in echolocation task, blind individuals show greater response bias when positioned closer to a lateral wall
Gori et al. [45]	B	Spatial & Temporal	Behavioral	19-62 years of age; 9 congenital blind individuals	See Vercillo et al. [51]; 1. Spatial bisection and minimum angle, 2. Pointing to single sound source, temporal bisection and slower version of temporal bisection	Blind individuals were impaired in auditory localization, but they showed no deficits in simpler auditory spatial tasks or auditory temporal bisection
Hüg et al. [53]	B	Spatial	Behavioral	7.1-37.3 months; 6 early (congenitally) blind infants	Minimum audible angle paradigms: 2 alternative forced choice: left/right of central sound (response: head turn, eye movement), single (control condition), lead and lag discrimination conditions (precedence effect conditions)	(Precedence effect: pair of sounds presented with brief delay from different locations, the first arriving sound has greater perceptual weight) no group difference in single condition, in lead and lag (precedence effect) condition the blind infants showed lower thresholds/better performance, blind infants used different localization behavior: facing object with ear
Kolarik et al. [38]	B	Spatial	Behavioral	21-86 years of age; 5 totally blind (from birth, 5, 9, or 19 years of age) and 6 partially sighted individuals	Distance discrimination in a virtual room<br><br>(sound sources simulated distances varied between 1 and 8 m)	Only totally blind (not partially sighted) individuals outperformed sighted participants by effectively using two auditory distance cues more efficiently
Kolarik et al. [49]	B	Spatial	Behavioral	25-69 years of age; 10 early onset blind individuals, lost sight before 5 years of age	Auditory distance judgment in far space, creating a virtual acoustic room (sound distances ranging from 1.22 to 13.79 m)	Blind individuals overestimated the auditory sources nearby and underestimated the auditory source in far distance

Study – Author(s)	Blind (B)/ Deaf (D)	Spatial/ Temporal/ Motion	Imaging technique/ Behavioral only	Blind/ Deaf participants (Age; onset of blindness/ deafness)	Task(s)	Results: Between group differences
Kolarik et al. [55]	B	Spatial	Behavioral	25-54 years of age (expert 20 years of age); 8 blind non-echolocators, 1 echolocation expert, blindness onset between birth and 5 years of age	Obstacle circumvention using echolocation (visual, auditory and tactile)	Blind non-echolocation experts navigated more effectively than blindfolded sighted participants using echolocation, echolocation expert performed similar or better than sighted individuals
Lewald [39]	B	Spatial	Behavioral	25-40 years of age; 5 blind individuals: 3 congenitally blind and 2 before the age of 5	Horizontal sound localization (head pointing to sound source; 21 loudspeakers), restrained vs. Not restrained head	Both groups performed approximately equal, blind individuals showed opposing systematic errors: suggesting blind participants differ mainly by perceptual mechanisms (enhancement of those) relating spatial coordinates to head and body rather than better discrimination of auditory cues
Lewald [42]	B	Spatial	Behavioral	25-40 years of age; 6 blind individuals: 3 congenitally blind and 3 before the age of 5	Vertical sound localization (11 loudspeakers)	In some blind individuals a deficit in absolute sound location detection in the vertical plane was shown, performance of relative position judgment did not differ
Nilsson and Schenkman [36]	B	Spatial	Behavioral	25-73 years of age;12 were blind from birth, 1 became blind at age 3, and 10 became blind after age 10	2 alternative-forced-choice task of left and right ear sound-discrimination using inter-aural time and level difference cues	More efficient use of inter-aural level difference cues, inter-aural time difference sensitivity was better than the one of age matched sighted controls but not than the one of younger sighted individuals
Schenkman and Nilsson [28]	B	Spatial	Behavioral	30-62 years of age; 7 congenitally blind individuals, 3 at 17, 18 or 38 years of age	2 alternative-forced-choice tasks of echolocation controlling for distance, signal duration and reverberation	Blind participants outperformed sighted, below 2 m distance both performed well, and the difference was greatest at 2 m distance echolocation

Study – Author(s)	Blind (B)/ Deaf (D)	Spatial/ Temporal/ Motion	Imaging technique/ Behavioral only	Blind/ Deaf participants (Age; onset of blindness/ deafness)	Task(s)	Results: Between group differences
Teng et al. [44]	B	Spatial	Behavioral	22-41 years of age; 6 blind, highly trained echolocators, blindness onset between birth and 17 years of age	2 alternative-force choice task of spatial discrimination using echolocation (relative position of two disks from each other)	No sighted control group, echolocation experts were able to discriminate horizontal offsets as small as ~1.2 degree auditory angle, strong correlation between echolocation acuity and age of blindness onset, precision comparable to that found in visual periphery of sighted individuals
Vercillo et al. [50]	B	Spatial	Behavioral	Mean age of 37.7 ± 5; 6 blind non-echolocation experts and 3 echolocation experts (aged 50, 54, and 57)	Space bisection task (sequence of 3 sounds – was second sound closer to first or third) and minimal audible angle task (23 locations of loudspeakers)	Blind individuals were impaired in the space bisection task, echolocation experts performed equally well/better than sighted; in minimum audible angle task the groups did not differ, although echolocation experts showed slightly better performance
Vercillo et al. [51]	B	Spatial & Temporal	Behavioral	9-14 years of age; 8 early (congenitally) blind individuals	1. Space bisection (sequence of 3 sounds – was second sound closer to first or third) and minimal audible angle task (23 loudspeakers), 2. Temporal bisection (3 sounds from central speaker, second sound temporally closer to either first or third)	Blind individuals performed worse than sighted in the spatial bisection and minimum audible angle task, blind individuals did not differ from sighted in the temporal bisection task
Vercillo et al. [52]	B	Spatial	Behavioral	22-56 years of age; 8 early blind individuals (all congenitally apart from 1 who lost vision at 10 years of age and for 1 unknown)	External spatial bisection task and body-centered frames of reference spatial bisection (18 loudspeakers, 3 sounds, second sound either temporally closer to first or third)	Blind individuals performed worse in the external spatial bisection task, but performance was similar to sighted in the body-centered reference frame task

Study – Author(s)	Blind (B)/ Deaf (D)	Spatial/ Temporal/ Motion	Imaging technique/ Behavioral only	Blind/ Deaf participants (Age; onset of blindness/ deafness)	Task(s)	Results: Between group differences
Voss et al. [46]	B	Spatial	Behavioral	21-55 years of age; 14 early blind individuals who lost their vision before the age of 11 years and 9 late blind individuals who lost their vision after the age of 16 years	Frontal minimum audible angle task, peripheral minimum audible angle task, minimum audible distance task (indicate whether second sound came from same or different position, judging relative position/distance)	All blind participants performed better when determining relative distance of sounds, also late blind participants outperformed sighted controls
Voss et al. [4]	B	Spatial	Behavioral	Mean age of 34.5 ± 12.11; 11 early blind individuals who lost their sight between 6 months ± 1 and a half a year	Binaural and monaural sound localization in vertical and horizontal plane (25 loudspeakers in the horizontal plane and 18 loudspeakers in the vertical plane)	Equal performance in binaural task in horizontal plane, in monaural task half of the blind individuals performed better than sighted individuals, some blind participants showed trade off in vertical and horizontal localization
Wallmeier and Wiegrebe [56]	B	Spatial	Behavioral	1 echolocation expert, congenitally blind	Virtual echo-acoustic space: using echolocation while in a corridor, 2 alternative, two-interval, forced-choice paradigm judging distance to frontal wall	Echolocation was possible over wide range of reference distances and environmental conditions and head rotations improved distance discrimination (note sighted were trained to perform well)
Zwiers et al. [41]	B	Spatial	Behavioral	23-42 years of age; 6 early blind individuals	2D sound localization in simple (no background noise) vs. complex scenes (with background noise coming from an array of 9 loudspeakers), target at any location in frontal hemi-field	Equal performance in simple scene, worse performance of blind individuals in more complex acoustic scene, worse performance in frontal region but superior in regions where vision is poor

Study – Author(s)	Blind (B)/ Deaf (D)	Spatial/ Temporal/ Motion	Imaging technique/ Behavioral only	Blind/ Deaf participants (Age; onset of blindness/ deafness)	Task(s)	Results: Between group differences
Anurova et al. [63]	B	Spatial	fMRI	34-58 years of age; 12 early blind individuals: 9 blind from birth and 3 by the second year of life	Relation of cortical thickness to sound localization, sound detection and discrimination (4 different locations in virtual auditory space)	No behavioral differences; activation during sound-localization and pitch-identification correlated negatively with cortical thickness in occipital areas of early blind participants, the following areas were involved: calcarine sulcus, lingual gyrus, superior and middle occipital gyri, and cuneus
Collignon et al. [3]	B	Spatial	TMS	22-55 years of age; 6 early (congenitally) blind	Localization judgement (2 alternative forced choice: left vs. right) while TMS over right intraparietal sulcus, the right dorsal occipital cortex and the right primary somatosensory cortex	TMS over right intraparietal sulcus (usually processing auditory spatial processing) did not affect blind participants' performance, TMS over right occipital cortex disrupted performance
Collignon et al. [61]	B	Spatial	fMRI	28-56 years of age; 11 early (congenitally) blind	Localization judgment (2 alternative forced choice: left vs. right of median plane)	No behavioral difference in binaural localization, notably likely due to stair-case procedure and absence of spectral cues for the sounds, blind participants recruited occipital cortex, right cuneus and right middle occipital gyrus, areas in dorsal occipital cortex involved in visuospatial/motion processing (possible attributable to the effect that fast presentation of sounds could be perceived as auditory motion) and part of network including regions for audiovisual spatial abilities
Collignon et al. [64]	B	Spatial	fMRI	22-60 years of age; 12 congenitally and 10 late blind individuals	Left vs. right ear sound source discrimination task	No behavioral difference (stair-case procedure), only blind individuals showed activation in occipital cortex, only early blind not late blind individuals showed activation in middle occipital gyrus and cuneus

Study – Author(s)	Blind (B)/ Deaf (D)	Spatial/ Temporal/ Motion	Imaging technique/ Behavioral only	Blind/ Deaf participants (Age; onset of blindness/ deafness)	Task(s)	Results: Between group differences
Garg et al. [60]	B	Spatial	fMRI	36-57 years of age; 9 congenitally blind	Covert attention switching to (endogenous) auditory targets task (verbal cues: "left", "right", "center")	No behavioral group difference, blind individuals showed stronger response to invalid targets in frontal eye field areas and those areas showed trend towards a response to center cues due to lack of visual input, bilateral activation in medial occipital cortex of blind individuals
Gougoux et al. [62]	B	Spatial	PET	22-48 years of age; 12 early blind individuals: 6 lost their vision at birth, 2 at 1 years of age, 1 at 2, 1 at 5, 1 at 8 and 1 at 14 years of age	Monaural and binaural sound localization (16 loudspeakers/9 speakers)	Increased activation in occipital cortex areas in early blind individuals that is linked to enhanced performance, lacking decrease of activation in visual cortex activity in blind individuals
Leclerc et al. [59]	B	Spatial	EEG	27 ± 5 years of age; 4 blind individuals (that showed best localization abilities in previous study)	Sound localization, 4 possible locations	Blind individuals more accurate in localizing binaural sounds (but not significantly), posterior shift of N1 (component influenced by stimulus features) and similar for P3 (attentional marker), both were larger in blind participants
Renier et al. [68]	B	Spatial	fMRI	34-58 years of age; 12 early blind individuals, congenitally blind or before the age of 2	3 Conditions: detection, identification (one back comparison) and spatial localization; stimuli were auditory (virtual auditory space) or vibro-tactile and varied in 2 dimensions: frequency and spatial location (4 sound sources)	No behavioral difference, middle occipital gyrus correlated with accuracy of individual sound localization, right middle occipital gyrus more activated during spatial than non-spatial tasks, thus the dorsal stream functional specialization of blind individuals appears to be maintained
Voss et al. [66]	B	Spatial	PET	22-54 years of age; 6 late blind individuals, onset of blindness ranged from 18 - 37 years	Monaural and binaural localization task (circular array of 9 loudspeakers)	No behavioral difference, sighted individuals showed reduced visual cortex activity, late blind individuals activated regions in occipital cortex (especially in the right hemisphere for binaural localization and bilaterally during monaural localization)

Study – Author(s)	Blind (B)/ Deaf (D)	Spatial/ Temporal/ Motion	Imaging technique/ Behavioral only	Blind/ Deaf participants (Age; onset of blindness/ deafness)	Task(s)	Results: Between group differences
Voss et al. [67]	B	Spatial	PET	Late blind: 33-54 years, early blind: 21-41 years (split into 2 early blind groups based on Gougoux et al. [62]: with and without superior performance); 6 late blind and 12 early blind individuals; onset of blindness ranged from 18 - 37 years	Monaural and binaural sound source discrimination task (circular array of 9 loudspeakers, same vs. different)	No behavioral difference in the binaural task, in the monaural task early blind individuals outperformed sighted and late blind participants, the better performance of early blind correlated with activity in left extrastriate, whereas activity in right ventral visual pathway in late blind individuals correlated negatively with performance
Wallmeier et al. [69]	B	Spatial	fMRI	48 and 33 years of age; 2 early blind echolocation experts	Echolocation (played own echolocations/ own vocalizations via headphones) in virtual echo-acoustic space specific in azimuth and distance	Pattern classification for left/right discrimination: In calcarine cortex for the echolocation experts (only significant for one), in sighted in the planum temporale
Weeks et al. [65]	B	Spatial	PET	Mean age of 42 years; 9 early (congenitally) blind individuals	Auditory localization (virtual sounds coming from 7 different azimuthal directions played via headphones)	No behavioral difference, blind and sighted participants activated posterior parietal areas, blind individuals additionally recruited right occipital cortex (originally intended for dorsal visual stream)
Lerens et al. [71]	B	Temporal	Behavioral	28-62 years of age; 14 early blind individuals, all being blind since birth or before 18 months of age	Duration discrimination task (same length or longer) and asynchrony detection task (synchronized or not with beat)	Early blind individuals had lower threshold for beat asynchrony detection, no difference in duration discrimination task
Lerens and Renier [70]	B	Temporal	Behavioral	24-60 years of age; 12 early blind individuals, 10 congenitally blind and 2 before the age of 3	Auditory target detection/ frequency discrimination	Blind individuals were faster when discriminating sounds in frontal and peripheral field, sighted participants discriminated frontal faster than peripheral sounds whereas there was no difference for speed of detection between central and spatial targets for blind individuals

Study – Author(s)	Blind (B)/ Deaf (D)	Spatial/ Temporal/ Motion	Imaging technique/ Behavioral only	Blind/ Deaf participants (Age; onset of blindness/ deafness)	Task(s)	Results: Between group differences
Weaver and Stevens [72]	B	Temporal	Behavioral	42-57 years of age; 10 early blind individuals, onset of blindness before the first year of life	Auditory gap detection	Gap detection thresholds of blind individuals were nearly identical to age matched sighted and slightly poorer than those of younger sighted individuals, yet non-significant
Stevens and Weaver [73]	B	Temporal	Behavioral	50.4 ± 5.9 years of age; 15 early (congenital) blind individuals	Auditory backward masking/recognition task: Temporal order judgment	Early blind performed better, significantly lower temporal order judgment threshold, advantages in perceptual consolidation
Roder et al. [77]	B	Spatial & Temporal	EEG/ERP	21-34 years of age; 8 congenitally blind participants	Spatial and temporal processing within stimulus selection task	The late amplitude of the auditory N1 was only modulated during temporal (not spatial) selection strategies in blind individuals, blind participants prefer temporal selection strategy; overall blind individuals performed better
Stevens et al. [75]	B	Temporal	fMRI	47.3 ± 10.5 years of age; 12 early blind participants	Auditory backward masking/recognition task: Temporal order judgment (same vs. different)	Early blind performed better than sighted individuals, activation in occipital areas (anterior calcarine sulcus) of early blind individuals correlated with performance
Van der Lubbe et al. [74]	B	Temporal	EEG	45-63 years of age; 12 early blind participants	Separate tactile and auditory duration discrimination tasks (preceding cues; valid/ invalid (cross-modal cue))	Early blind individuals responded faster and more accurate (duration discrimination more difficult for tactile than auditory), posterior negativity N1 component, enhanced occipital negativity for the blind relative to the sighted (comparable in auditory and tactile task), no difference in cueing effects

Study – Author(s)	Blind (B)/ Deaf (D)	Spatial/ Temporal/ Motion	Imaging technique/ Behavioral only	Blind/ Deaf participants (Age; onset of blindness/ deafness)	Task(s)	Results: Between group differences
Cappagli and Gori [78]	B	Motion	Behavioral	Blind children: 9-17 years, children with low vision: 7-17 years, early blind adults: 20-54 years, and late blind adults: 20-63 years of age; 10 blind and 10 low vision children, and 11 blind adults	Static and dynamic sound localization task (23 loudspeakers in straight line on horizontal plane)	No difference between children with low vision and blindness and late and early blind adults, contrary to blind adults, children with low vision and blindness were impaired in sound localization, adult like performance was achieved around 13 years of age
Cappagli et al. [79]	B	Motion	Behavioral	3-4 years of age; 5 low vision, 4-5 years of age; 2 blind individuals	Static and dynamic sound localization task (23 loudspeakers in straight line on horizontal plane)	Worse performance of blind children compared to those with low vision in both tasks
Finocchietti et al. [83]	B	Motion	Behavioral	20-65 years of age; 12 congenitally blind and 8 late blind participants (loss of vision after 10 to 40 years of age)	Complex sound motion detection	Early blind individuals show impairment in lower plane, whereas sighted as well as late blind individuals do not show the deficit
Guerreiro et al. [84]	B	Motion	Behavioral	16-43 years of age; 6 individuals with partially restored vison (bilateral cataract-reversal at the age of 5-24 months)	Cross-modal motion after-effects: Experiment 1: Audition to vision: Judging size of visually changing stimulus after adapting (increasing in sound pressure level) auditory stimulus was presented; Experiment 2: Vision to audition: Judging auditory loudness change after visually changing stimulus was shown	Only cataract-reversal participants’ visual perception was influenced by adaptation to preceding auditory motion (visual motion aftereffect), sighted and cataract-reversal individuals showed an auditory motion aftereffect (auditory stimulus perception was affected by visual motion)

Study – Author(s)	Blind (B)/ Deaf (D)	Spatial/ Temporal/ Motion	Imaging technique/ Behavioral only	Blind/ Deaf participants (Age; onset of blindness/ deafness)	Task(s)	Results: Between group differences
Lewald [80]	B	Motion	Behavioral	22-46 years of age; 7 early blind individuals: 6 congenitally blind and 1 under 6 months of age, 7 late blind: blind between the age of 5 and 32 years	Stationary and moving sound detection (semicircular horizontal loudspeaker array ranging from −90° to 90°/ left to right side in steps of 2°)	No difference in performance in stationary sound detection but (early and late) blind individuals outperform sighted participants in moving sound detection
Vercillo et al. [82]	B	Motion	Behavioral	22-56 years of age; 8 congenitally blind participants	Simple pointing task with static sound sources and sound localization task with moving sounds (once with/without moving head; 8 different positions)	Blind individuals showed left-wards bias for localization of static and a minor bias for moving sounds, in contrast to sighted, their localization abilities were affected by head motion, likely blind individuals have body-centered spatial representations
Yabe and Kaga [81]	B	Motion	Behavioral	12-26 years of age; 14 congenitally blind participants, 9 acquired blindness, 14 individuals with residual vision	Sound lateralization, sound image moving to the right or left side: 2 alternative forced choice	Blind subjects did not show better pure tone audiometric thresholds but better sound lateralization/inter-aural time difference discrimination than sighted (and those with residual vision)
Bedny et al. [85]	B	Motion	fMRI	Early blind: 37-61 years, 6 late blind individuals: 43-53 years of age; 10 early (congenitally) blind, 5 late blind who lost their vision between 9 to 34 years of age, 1 early blind who lost vision between the age of 2-3	Movement differentiation (footsteps or tones away or towards the participant)	No difference in accuracy but late blind were slower than sighted, MT/MST recruited in early blind but not in late blind or sighted individuals (and not in 1 individual who lost vision between 2-3 years of age), all blind participants showed reduced functional connectivity between MT/MST and other visual regions but increased connectivity with lateral prefrontal areas (early blindness affects functions of feedback projections from prefrontal cortex)

Study – Author(s)	Blind (B)/ Deaf (D)	Spatial/ Temporal/ Motion	Imaging technique/ Behavioral only	Blind/ Deaf participants (Age; onset of blindness/ deafness)	Task(s)	Results: Between group differences
Dormal et al. [91]	B	Motion	fMRI	23-62 years of age; 15 early blind individuals (12 congenitally and 3 <= 1 year of age)	Auditory motion processing (in-depth, laterally moving (left to right ear) and static sounds)	Groups did not differ in accuracy, right hMT+/V5 and V3A take over auditory motion processing in early blind individuals, reduced selectivity for auditory motion in planum temporale (usually processing auditory motion in sighted individuals)
Jiang et al. [92]	B	Motion	fMRI	31-63 years of age; 7 early congenitally blind individuals/ lost sight before 1.5 years of age	Classification of direction of motion based on BOLD responses (simulating motion by using changes in inter-aural time/level difference and Doppler shift)	Early blind outperformed sighted individuals, direction of the auditory motion can be discriminated within hMT+ in blind and in right planum temporale in sighted individuals
Jiang et al. [93]	B	Motion	fMRI	31-63 years of age; 7 early blind individuals (congenitally or before/at 5 years of age), 4 late-blind (lost vision between the age of 34 and 59 years of age), 1 sight recovery participant (blind at the age of 3 and gained sight by the age of 46, now 60 years of age)	Classification of direction of motion based on BOLD responses (by classifier deciding on basis of training data), same paradigms as in Jiang et al. [92]	Early blind individuals outperformed sighted participants, cortical reorganization observed is entirely developmental and permanent/ irreversible, in late blind participants during auditory motion: no activation of hMT+ and no loss of activation in right planum temporale
Saenz et al. [94]	B	Motion	fMRI	53 years if age; 2 partial sight recovery individuals	Inter-aural level / inter-aural time difference auditory motion detection vs. control auditory stimuli (added duration judgement to keep tasks demands comparable)	Recruitment of MT+ in sight recovery individuals specifically during auditory motion (not during another auditory stimulus presentation)

Study – Author(s)	Blind (B)/ Deaf (D)	Spatial/ Temporal/ Motion	Imaging technique/ Behavioral only	Blind/ Deaf participants (Age; onset of blindness/ deafness)	Task(s)	Results: Between group differences
Bottari et al. [95]	B	Motion	EEG	9-35 years of age; 12 sight recovery participants (tested after 12 months to 33 years after cataract removal)	Visual and auditory global motion task	Sight-recovery participants showed worse performance during the visual global motion, yet, better performance during the auditory global motion task than sighted and visually impaired individuals; reduced alpha oscillations during visual task and enhanced beta oscillations during auditory task in sight-recovered participants
Leclerc et al. [58]	B	Motion	EEG	8 early (congenitally) blind individuals (short description due to short report)	Sound localization, 4 possible locations	Blind and sighted subjects did not differ in overall EEG power in any frequency range, yet, EEG coherence between fronto-central and occipital sites was increased in blind individuals in theta, alpha, and beta frequencies, in addition posterior shift, supports idea that occipital region usually reserved for visual functions is more integrated into auditory attention/processing
Lewald and Getzmann [90]	B	Motion	EEG/ERP, sLORETA	24-42 years of age; 8 blind individuals (4 congenitally or early blind (before 6 months of age) and 4 late blind with onset between 5 and 22 years of age)	Sound motion detection (–90° to +90°/ left to right in steps of 2°)	Behavioral findings based on Lewald [80]: Better auditory motion perception in blind individuals requiring only half the minimum audible angle of sighted, cN1 ERP component twice as the amplitude of sighted, and stronger activation in blind participants in ventral visual regions of the right visual cortex and middle temporal area V5
Lewis et al. [86]	B	Motion	fMRI	32-56 years of age; 7 early blind participants, onset of blindness at birth or till 18 months of age	3 auditory tasks (auditory letters, auditory motion (same or different motion of two stimuli), and auditory frequency discrimination); (and 3 tactile tasks)	No behavioral difference, higher recruitment of occipital cortex in blind individuals across tasks, unselective response (unspecific to task) might represent an additive shift, although skill enhancement was found in past studies rather specific in nature

Study – Author(s)	Blind (B)/ Deaf (D)	Spatial/ Temporal/ Motion	Imaging technique/ Behavioral only	Blind/ Deaf participants (Age; onset of blindness/ deafness)	Task(s)	Results: Between group differences
Poirier et al. [87]	B	Motion	fMRI	18-68 years of age; 6 early blind individuals, onset of blindness between birth and 18 months of age	Movement detection (left to right or right to left side, simulated by changing inter-aural level difference, static sounds: sensation as if presented at 8 different locations)	No behavioral difference, recruitment of motion processing areas (bilateral dorsal and ventral premotor cortex, the left inferior parietal lobule, the right V5 area, the bilateral V3/V3A area) during auditory motion processing and additionally bilateral V1/V2 in the blind individuals only (a priori selection of brain regions)
Strnad et al. [88]	B	Motion	fMRI;	37-61 years of age; 10 early blind individuals (same as in Bedny et al. [85]	Movement differentiation (footsteps or tones away or towards the participant); see Bedny et al. [85]	Behavioral results reported in Bedny et al. [85], MT+ (bilateral but mainly right hemisphere) recruitment for auditory motion, differentiated between high and low motion (footsteps vs. sounds), yet, MT+ also sensitive to motion information in sighted individuals
Thaler et al. [30]	B	Motion	fMRI	29, 42 and 44 years of age; 3 blind echolocation experts (lost sight at birth, at 1 or at 3 years of age)	Moving echolocation detection task (recorded echo/vocalizations played via headphones), comparing stationary vs. moving sounds (and visual motion)	Region of interest (temporal-occipital areas) dissociation in both groups of echo-motion and source motion, contra-lateral motion preference for echo-motion only in blind subjects, blind echolocation experts outperformed sighted in echolocation-based motion task only
Cattaneo et al. [110]	D	Spatial	Behavioral	16-68 years of age; 24 deaf signers, profoundly deaf (>96 dB)	Line bisection task	Compared to hearing (non)signers, deaf individuals did not show leftward bias
Chen et al. [98]	D	Spatial	Behavioral	Mean age of 20 ± 1.5 years; 20 congenitally and genetically deaf individuals, binaural hearing loss above 90 dB	Flanker task: is certain shape present in one of 6 locations around center	Higher influence of distractors in periphery in near space and central distractors in far space in deaf individuals, suggested reorganization of attention in near and far space

Study – Author(s)	Blind (B)/ Deaf (D)	Spatial/ Temporal/ Motion	Imaging technique/ Behavioral only	Blind/ Deaf participants (Age; onset of blindness/ deafness)	Task(s)	Results: Between group differences
Codina et al. [99]	D	Spatial	Behavioral	Children: 5-15 years of age; 25 deaf participants that were diagnosed with deafness within the first 2 years of life; adults: 18-45 years of age; 17 deaf adults (amount of hearing loss and age of deafness in adults not mentioned)	Visual field test: Detection of location of flash light	5 to 10 years olds reacted slower and less accurate when detecting dim light in far peripheral field, performance equal to hearing children at the age of 11-12 years, aged 13-15 deaf individuals outperform hearing individuals in periphery, adult-like performance of both, deaf and hearing participants at the age of 11-12
Codina et al. [108]	D	Spatial	Behavioral	18-45 years of age; 17 profoundly deaf individuals, either deaf from birth or onset before 8 months	Forced choice visual detection paradigm in far periphery	Faster reaction times of deaf individuals to stimuli in periphery, faster than hearing-signers who outperformed non-signers
Daza and Phillips-Silver [104]	D	Spatial	Behavioral	6-12 years of age, 56 deaf children with hearing loss before the age of 2 (50 % congenitally hearing loss), degree of hearing loss varied between mild to profound with more than 75 % having severe or profound hearing loss	Child version of attention network test (ANT), spatial attention orienting (cost/benefit cueing paradigm with left/right discrimination)	Alerting was affected by deafness: slower RTs, enhanced moving and engaging (orienting mechanisms, faster orienting), no group differences in executive functioning (note, findings based on comparison of high and low auditory simulation: Children with CI and wo used oral language compared to children without a CI who mainly utilized sign language)
Dye et al. [101]	D	Spatial	Behavioral	Experiment 2: deaf children aged 7-17 years of age (3 age groups with a total of 49 children), unaided hearing loss of 70 dB or higher in the better ear	Useful field of view task (UFOV) (central and peripheral task), testing visual attention in the functional visual field (no head/eye movements)	Enhanced performance of deaf children only after being 11 years old, equal group performance at the age of 7-10 years
Dye and Hauser [102]	D	Spatial	Behavioral	6-8 years of age (12 deaf individuals), 9-13 years of age (25 deaf individuals); severe-to-profound hearing loss of above 75 dB in the better ear	Sustained attention, selective attention, and cognitive control using the vigilance and distractibility forms of the GDS CPT	No group differences in sustained attention, younger deaf individuals (before 9 years of age) more distracted by peripheral stimuli, deaf children showed higher commission error rate during selective attention

Study – Author(s)	Blind (B)/ Deaf (D)	Spatial/ Temporal/ Motion	Imaging technique/ Behavioral only	Blind/ Deaf participants (Age; onset of blindness/ deafness)	Task(s)	Results: Between group differences
Dye [105]	D	Spatial	Behavioral	18-40 years of age; 16 deaf participants, deaf from birth or during the first 6 months, severe-to-profound hearing loss of 75 dB or greater	3 versions of the useful field of view task (UFOV) task: Central only, central and peripheral, duals task with distractors, testing visual attention in the functional visual field (no head/eye movements)	Equal performance in central identification only, no group difference in central and peripheral task and not affecting central discrimination task, performance on foveal task was impaired by peripheral attention task even more in deaf individuals
Heimler et al. [112]	D	Spatial	Behavioral	24-44 years of age; 14 deaf individuals, hearing loss at birth or until 3 years of age, hearing loss of 70 dB or more in better ear	Gaze cueing task, peripheral discrimination task	Less consistent gaze cueing of deaf individuals, comparable arrow-cueing so that effect of gaze cueing specific to nature of stimulus (gaze)
Heimler et al. [113]	D	Spatial	Behavioral/Eye-tracking	22-43 years of age; 20 deaf individuals, diagnosis between birth and 3 years of age, above 70 dB hearing loss in better ear	Overt saccadic target-selection task (participants have to search for a target in between other stimuli and distractor target)	Deaf individuals were slower at initiating first saccadic responses and influenced by saliency of target (manipulated by color); data modelling showed that the slower saccades likely led to the reduced effect of saliency (no linguistic measure explained this effect)
Netelenbos and Savelsbergh [103]	D	Spatial	Behavioral	2 age groups: 5-7 years and 10-12 years; 50 deaf children, congenital hearing loss of 90 dB loss or greater	Localization of visual targets within and beyond field of view/visual search	Deaf children show higher tendency to use slow-acting search strategies/ performance poorer
Prasad et al. [106]	D	Spatial	Behavioral/ Occulo-motor response	24.2 ± 6.46 years of age; 15 congenitally deaf individuals	Attention orienting: Spatial cueing task	Deaf individuals showed (marginally) higher cueing effect for ocular responses

Study – Author(s)	Blind (B)/ Deaf (D)	Spatial/ Temporal/ Motion	Imaging technique/ Behavioral only	Blind/ Deaf participants (Age; onset of blindness/ deafness)	Task(s)	Results: Between group differences
Proksch and Bavelier [107]	D	Spatial	Behavioral	Mean age of 21.2 years of age; 10 deaf students, congenital hearing loss of 85 dB or more	Detection of target stimulus out of several stimuli under simultaneous presentation of distractor stimulus	Deaf participants payed more attention to periphery and less to the central field and it could not be attributed to sign language only, thus likely a result of auditory deprivation
Rothpletz et al. [111]	D	Spatial	Behavioral	18-45 years of age; 10 severe-to-profoundly deaf individuals, hearing loss greater than 80 dB in both ears with age of onset of hearing loss ranging from birth to 18 months of age	Peripheral visual orienting task (non-distractor and distractor condition), response: initiating head turns	Slower responses of deaf individuals compared to hearing to stimuli in near periphery (and near and distant periphery with distractors)
Sladen et al. [109]	D	Spatial	Behavioral	21-45 years of age; 10 deaf individuals, hearing loss prior to 2 years of age, hearing loss of > 80 dB for each ear	Flanker task, reaction to irrelevant competing stimuli has to be inhibited to perform well (spatial attention test)	Faster reaction times of hearing compared to deaf individuals, yet, hearing individuals made more errors, deaf individuals had a significantly greater interference effect at para-foveal eccentricity, wider spatial attention allocation of visual resources
Seymour et al. [6]	D	Spatial	EROS recordings	18-47 years of age; 10 deaf individuals (7 congenitally and 3 hearing loss between 1-3 years of life), binaural hearing loss in better ear greater than 80 dB	Useful field of view task (UFOV), testing visual attention in the functional visual field (no head/eye movements)	Lower detection threshold of deaf individuals in peripheral UFOV task, linked to activation in Brodmann area 22 (right hemisphere), and a differing activation pattern in visual cortex regions (deaf individuals upregulate activity more than normal hearing individuals)
Heming and Brown [117]	D	Temporal	Behavioral	18-31 years of age; 10 deaf individuals, 8 were congenitally deaf and 2 before the age of 2	Judging simultaneousness of visual and tactile stimuli	Higher visual (and tactile) temporal threshold, i.e., slower reaction times, yet, reaction times of deaf individuals were not affected by spatial location

Study – Author(s)	Blind (B)/ Deaf (D)	Spatial/ Temporal/ Motion	Imaging technique/ Behavioral only	Blind/ Deaf participants (Age; onset of blindness/ deafness)	Task(s)	Results: Between group differences
Nava et al. [76]	D	Temporal	Behavioral	18-40 years of age; 10 deaf individuals, 2 congenitally, 3 deaf before age of 2, 5 between 2-4 years of age, bilateral profound hearing loss above 85 dB	Visual temporal order judgment of visual stimuli pairs in varying locations	Deaf individuals showed faster discrimination of temporal order, especially when first stimulus appeared in peripheral field
Iversen et al. [116]	D	Temporal	Behavioral	Mean age of 28.9 years; 23 deaf individuals, 20 congenitally deaf and 3 became deaf before the age of 3, 3 with hearing loss larger than 70 dB and 20 with hearing loss larger than 90 dB	Visual and auditory finger tapping task to align with discrete point in time (no continuous movement) to test whether visual synchronization can get as accurate as auditory	Deaf participants showed better synchronization with visual stimuli compared to hearing subjects that generally are better in the case of auditory stimuli being presented
Kowalska and Szelag [120]	D	Temporal	Behavioral	Mean age of 17.5 years ± 9; 16 deaf individuals, all being congenitally deaf and having a hearing loss equal to or above 90 dB	Reproduction and production of durations a visual stimulus had to appear on a screen	Deafness affects duration judgment of visual stimuli: overestimating durations shorter than 2 s and underestimate those above 3 s
Bola et al. [22]	D	Temporal	fMRI	Mean age of 27.6 ± 4.51 years; 15 congenitally deaf adults, either genetic or pregnancy-related hearing loss being deaf from birth, hearing aid use by most of them with broadly varying speech-understanding; mean hearing loss of 98 dB for the left ear and 103 dB for the right ear	Visual and auditory rhythm discrimination task	No behavioral difference – staircase procedure; deafness leads to increased connectivity between auditory cortex and dorsal occipital cortex, activation of posterior-lateral part of auditory cortex in deaf individuals during visual rhythm discrimination mirroring the activation pattern in hearing subjects during auditory rhythm discrimination

Study – Author(s)	Blind (B)/ Deaf (D)	Spatial/ Temporal/ Motion	Imaging technique/ Behavioral only	Blind/ Deaf participants (Age; onset of blindness/ deafness)	Task(s)	Results: Between group differences
Almeida et al. [127]	D	Motion	Behavioral	18-40 years of age; 14 deaf individuals (13 congenitally deaf, 1 hearing loss at 3 years of age); 8 had binaural hearing loss from 71-90 dB and 4 had binaural hearing loss above 91 dB	Direction of motion discrimination task (moving random-dot patterns to test differential behavioral pattern in vertical vs. horizontal visual plane)	Deaf individuals showed better motion detection abilities than hearing individuals only in the horizontal plane: while hearing individuals show no difference in motion detection between locations, deaf individuals were better at discriminating motion in the horizontal compared to the vertical plane
Bosworth and Dobkins [130]	D	Motion	Behavioral	Mean age of 31.1 years; 16 deaf participants, 12 deaf since birth, 2 acquired deafness due to illness, 2 unaware of cause, binaural hearing loss of at least 80 dB	Direction of motion discrimination paradigm (left vs. right)	Better orienting and selective attentional processes in deaf individuals (no differences in divided attention), most likely the result of auditory deprivation rather than sign language
Bosworth and Dobkins [128]	D	Motion	Behavioral	Mean age of 31.1 years; 16 deaf participants, 12 deaf since birth, 2 acquired deafness due to illness, 2 unaware of cause, binaural hearing loss of at least 80 dB	Direction of motion detection (left vs. right)	Inferior visual field advantage and improved performance in periphery linked to deafness, whereas right visual field advantage linked to sign language experience, processed in the left hemisphere
Bosworth et al. [129]	D	Motion	Behavioral	Mean age of 26 ± 1.9 years; 9 early deaf adults, all congenitally deaf except for one person who was deaf from 15 months of age, binaural hearing loss of above 80 dB	Investigation of attention and laterality in motion, form and brightness discrimination tasks, variability of attention in conditions	Right visual field advantage in deaf signers in motion, form processing but not for brightness task, not attention and modulatory effects of attention suggesting early sensory changes
Brozinsky and Bavelier [124]	D	Motion	Behavioral	Mean age of 20.5 years; 13 deaf individuals, binaural hearing loss of at least 75 dB (onset of hearing loss not mentioned)	Velocity detection task within spatial four-alternative forced choice	Right visual field advantage, no superior performance in periphery in low level motion

Study – Author(s)	Blind (B)/ Deaf (D)	Spatial/ Temporal/ Motion	Imaging technique/ Behavioral only	Blind/ Deaf participants (Age; onset of blindness/ deafness)	Task(s)	Results: Between group differences
Hauthal et al. [125]	D	Motion	Behavioral	32-60 years of age; 19 deaf participants, 14 deaf since birth, 5 deaf before age of 7, binaural hearing loss of at least 90 dB	Movement localization task and investigation of motion direction (moving dots, left/right)	Deaf participants showed left visual field advantage during movement localization, faster and more accurate reaction when detection direction of visual motion
Shiell et al. [21]	D	Motion	Behavioral	Mean age of 31.2 years; 16 deaf subjects, 7 congenitally deaf, 2 deafened from illness at 6 and 11 months of age, binaural hearing loss above 90 dB	Visual motion detection task (moving gratings, left or right one moving)	Deaf participants showed lower movement detection thresholds
Bavelier et al. [131]	D	Motion	fMRI	18-27 years of age; 11 (genetically) congenitally deaf individuals, binaural hearing loss above 90 dB	Luminance and velocity task, detect dimming and velocity changes of dots including different spatial attention manipulations	Deaf individuals performed better in periphery, hearing individuals better in central field; enhanced recruitment of motion selective region MT-MST during peripheral motion recognition due to auditory deprivation larger recruitment of motion areas in left hemisphere related to sign language
Finney et al. [135]	D	Motion	MEG	Mean age of 30 ± 4.9 years; 5 congenitally deaf	Movement differentiation (left vs. right)	(Note: Movement differentiation was mainly just to ensure attention), auditory cortex involvement in deaf individuals during first few hundred milliseconds of presentation of auditory movement, early response suggesting direct projection from visual thalamus to primary auditory cortex
Shibata et al. [136]	D	Motion	fMRI	19-32 years of age; 6 deaf individuals, hearing loss in the better ear was greater than 95 dB	Motion in the periphery: 2 alternative force choice movement detection (additionally shape matching, mental rotation task)	(Note: Checked that subjects perform at nearly 100% accuracy), right temporal lobe recruitment in deaf individuals, effect was greater for movement task, especially in the right superior and middle temporal gyrus

Study – Author(s)	Blind (B)/ Deaf (D)	Spatial/ Temporal/ Motion	Imaging technique/ Behavioral only	Blind/ Deaf participants (Age; onset of blindness/ deafness)	Task(s)	Results: Between group differences
Shiell and Zatorre [134]	D	Motion	fMRI	21-37 years of age; 11 deaf individuals, 10 congenitally, 1 deaf at 6 months, hearing loss greater than 90dB in both ears	Visual motion detection task (moving gratings – moving speed adjusted by adaptive staircase procedure, 2 alternative forced choice, left or right one moving)	(Note: No sighted control group) activation of right planum temporale correlates with visual motion detection, measures indicate altered myelination/axon density
Vachon et al. [137]	D	Motion	fMRI	21-52 years of age; 16 congenitally or pre-lingual deaf individuals, profound deafness of => 90dB	Visual motion detection task (difference in motion coherence) up vs. downward perceived motion	Main interest in ventral visual pathway, no difference between deaf and hearing individuals when comparing motion to static condition but when comparing motion and static condition against baseline: increased activation in (especially right) superior temporal gyrus and area at the junction of parieto-occipital sulcus and calcarine fissure in deaf, cross-modal recruitment of ventral pathway not specialized for motion

Abbreviations: B, Blind, D, Deaf, CI, cochlear implant, fMRI, functional magnetic resonance imaging, PET, positron-emission tomography, EEG, electroencephalography, ERP, event-related potential, TMS, transcranial magnetic stimulation, middle temporal complex (hMT+), middle temporal (MT) and medial superior temporal (MST), MEG, magnetoencephalography.
